# *Drosophila* Abi maintains blood cell homeostasis by promoting clathrin-mediated endocytosis of Notch

**DOI:** 10.1083/jcb.202505091

**Published:** 2025-12-26

**Authors:** Hyun Gwan Park, Seunghwan Song, Joohyung Kim, Seungbok Lee

**Affiliations:** 1 Department of Cell and Developmental Biology and Dental Research Institute, Seoul National University, Seoul, Korea; 2 Interdisciplinary Program in Neuroscience, Seoul National University, Seoul, Korea

## Abstract

Abl-interactor (Abi) proteins induce actin polymerization by activating Wiskott–Aldrich syndrome protein (WASp) or SCAR/WASP-family verprolin-homologous protein. Loss of mammalian Abi1 causes myeloproliferative neoplasm; however, little is known about how the Abi family of actin-regulatory proteins regulates blood cell homeostasis. Here, we demonstrate that *Drosophila* Abi promotes plasmatocyte-to-crystal cell transdifferentiation but represses plasmatocyte-to-lamellocyte transdifferentiation through Notch signaling. Consistent with a previously demonstrated role of clathrin-mediated endocytosis (CME) in Notch signaling activation, we find that Abi promotes Notch-CME by recruiting WASp and the Notch receptor to nascent sites of CME. Finally, we demonstrate that CME and crystal cell formation are inhibited by Abelson (Abl)-mediated phosphorylation of Abi but require PTP61F, a phosphatase that reverses this phosphorylation. Our findings identify Abi as a critical integrator of actin remodeling and Notch-CME and reveal opposing roles of Abl and PTP61F in regulating Abi activity to maintain blood cell homeostasis.

## Teaser

Park et al. show that *Drosophila* Abi, a regulator of actin cytoskeletal dynamics, controls blood cell homeostasis by activating Notch signaling through clathrin-mediated receptor endocytosis. This regulatory process is promoted by PTP61F-mediated Abi dephosphorylation but antagonized by Abl-mediated Abi phosphorylation, revealing a reversible phosphorylation switch that integrates actin remodeling, endocytosis, and Notch signaling.

## Introduction

Abl-interactor (Abi) proteins were originally identified as binding partners and substrates of the Abelson (Abl) family of tyrosine kinases (Dai and Pendergast, 1995; Juang and Hoffmann, 1999; Shi et al., 1995), whose deregulated activation through chromosomal translocation is linked to chronic myeloid leukemia and a subset of acute lymphocytic leukemia in humans (Burmeister et al., 2008; Sawyers, 1999). Despite this biochemical relationship, the role of Abi proteins in blood cell development remains unclear. Loss of Abi1 in a murine pro-B-cell line impairs Bcr-Abl–induced actin remodeling, cell migration, and leukemogenesis (Faulkner et al., 2020; Sun et al., 2008), suggesting that Abi1 mediates oncogenic Abl signaling. In contrast, other studies identified human Abi1 as a recurrent fusion partner of mixed-lineage leukemia, frequently disrupted by chromosomal translocation in acute myeloid leukemia (Morerio et al., 2002; Shibuya et al., 2001; Taki et al., 1998). Furthermore, bone marrow–specific knockout of *Abi1* in mice causes myeloproliferative neoplasm (Chorzalska et al., 2018), supporting a tumor suppressor function. These apparently contradictory findings imply that Abi acts through Abl-dependent and Abl-independent mechanisms.

Abi proteins are multimodular proteins with an N-terminal SCAR/WASp-family verprolin-homologous protein–binding WAB domain, a central Kette/Nap1-binding homeodomain homologous region, and a C-terminal Abl-binding Src homology 3 (SH3) domain (Dai and Pendergast, 1995; Echarri et al., 2004; Juang and Hoffmann, 1999; Ryu et al., 2009; Shi et al., 1995). By scaffolding SCAR and Kette, Abi organizes proper assembly of the SCAR complex (Eden et al., 2002; Gautreau et al., 2004; Innocenti et al., 2004), which relays Rac1 signaling to the actin-nucleating Arp2/3 complex (Rotty et al., 2013). In *Drosophila*, Abl-mediated phosphorylation of Abi is required for Rac1- and SCAR-dependent lamella formation and macropinocytosis (Huang et al., 2007; Kim et al., 2019), linking Abl signaling to actin-dependent cellular processes (Sato et al., 2012). Intriguingly, the SH3 domain of Abi also binds and activates the Wiskott–Aldrich syndrome protein (WASp), another Arp2/3 activator required for vesicular transport in mammalian cells and bristle formation in *Drosophila* (Bogdan et al., 2005; Innocenti et al., 2005). However, how Abl activation impacts Abi-WASp–dependent cellular processes remains unclear, as does the mechanism by which actin-regulatory Abi proteins act as tumor suppressors in blood cell development.

To address these questions, we used the *Drosophila* hematopoietic system, which comprises three classes of terminally differentiated cells (collectively called hemocytes): plasmatocytes performing phagocytic function, crystal cells mediating melanization, and lamellocytes involved in parasitoid encapsulation and killing (Banerjee et al., 2019; Letourneau et al., 2016). During larval development, hemocytes originate from two lineages: the lymph gland (LG) and the embryonic lineage, which colonizes the circulation and sessile patches underneath the cuticle (referred to as the peripheral compartment) (Holz et al., 2003; Jung et al., 2005; Makhijani et al., 2011). In the LG, mature hemocytes arise mainly from progenitor cells (prohemocytes) in the inner medullary zone and subsequently populate the peripheral CZ (Banerjee et al., 2019; Jung et al., 2005; Letourneau et al., 2016). Recent studies have shown that LG crystal cells can also be produced directly from mature plasmatocytes through transdifferentiation (Marcetteau et al., 2025). In contrast to largely progenitor-based LG hematopoiesis, larval peripheral hematopoiesis primarily relies on the self-renewal of Hemolectin-positive (Hml^+^) mature plasmatocytes (Makhijani et al., 2011) and their transdifferentiation into crystal cells or lamellocytes (Anderl et al., 2016; Cevik et al., 2019; Csordás et al., 2021; Honti et al., 2010; Leitao and Sucena, 2015; Stofanko et al., 2010).

Notch signaling is required for both progenitor-based and transdifferentiation-dependent formation of crystal cells during larval development (Blanco-Obregon et al., 2020; Duvic et al., 2002; Lebestky et al., 2003; Leitao and Sucena, 2015; Marcetteau et al., 2025), yet the molecular mechanisms that precisely regulate Notch-dependent hematopoiesis remain poorly understood. In nonhematopoietic contexts, Abl kinase negatively regulates Notch by promoting endocytic clearance of the receptor (Miranda-Alban et al., 2025; Xiong et al., 2013), suggesting that Abl and its downstream effectors may similarly influence Notch signaling during hematopoiesis.

Here, we investigated the role of *Drosophila* Abi in peripheral and LG hemocyte development, focusing on its opposing regulation by Abl and the *Drosophila* ortholog of mammalian PTP1B (PTP61F), which reverses Abl-mediated phosphorylation of Abi (Huang et al., 2007). We demonstrate that Abi promotes plasmatocyte-to-crystal cell transdifferentiation but represses plasmatocyte-to-lamellocyte conversion through Notch signaling. Clathrin-mediated endocytosis (CME) is required for ligand-induced Notch signaling (Chapman et al., 2016; Windler and Bilder, 2010). Our data indicate that Abi activates Notch signaling by promoting CME in hemocytes, a function that depends on its interaction with WASp but not SCAR. We further show that Abi promotes Notch-CME through a direct interaction with Notch, which is enhanced by Ser-induced Notch ubiquitination. Finally, we provide evidence that Notch-CME and crystal cell differentiation are inhibited by Abl-mediated Abi phosphorylation but require PTP61F-mediated Abi dephosphorylation. Together, these findings identify Notch-CME as a central mechanism through which the Abi family of actin-regulatory proteins controls blood cell development, and highlight the opposing roles of Abl and PTP61F in modulating Abi-dependent Notch-CME and signaling.

## Results

### *Drosophila* Abi is required in differentiated plasmatocytes for hematopoietic homeostasis

**Figure S1.**
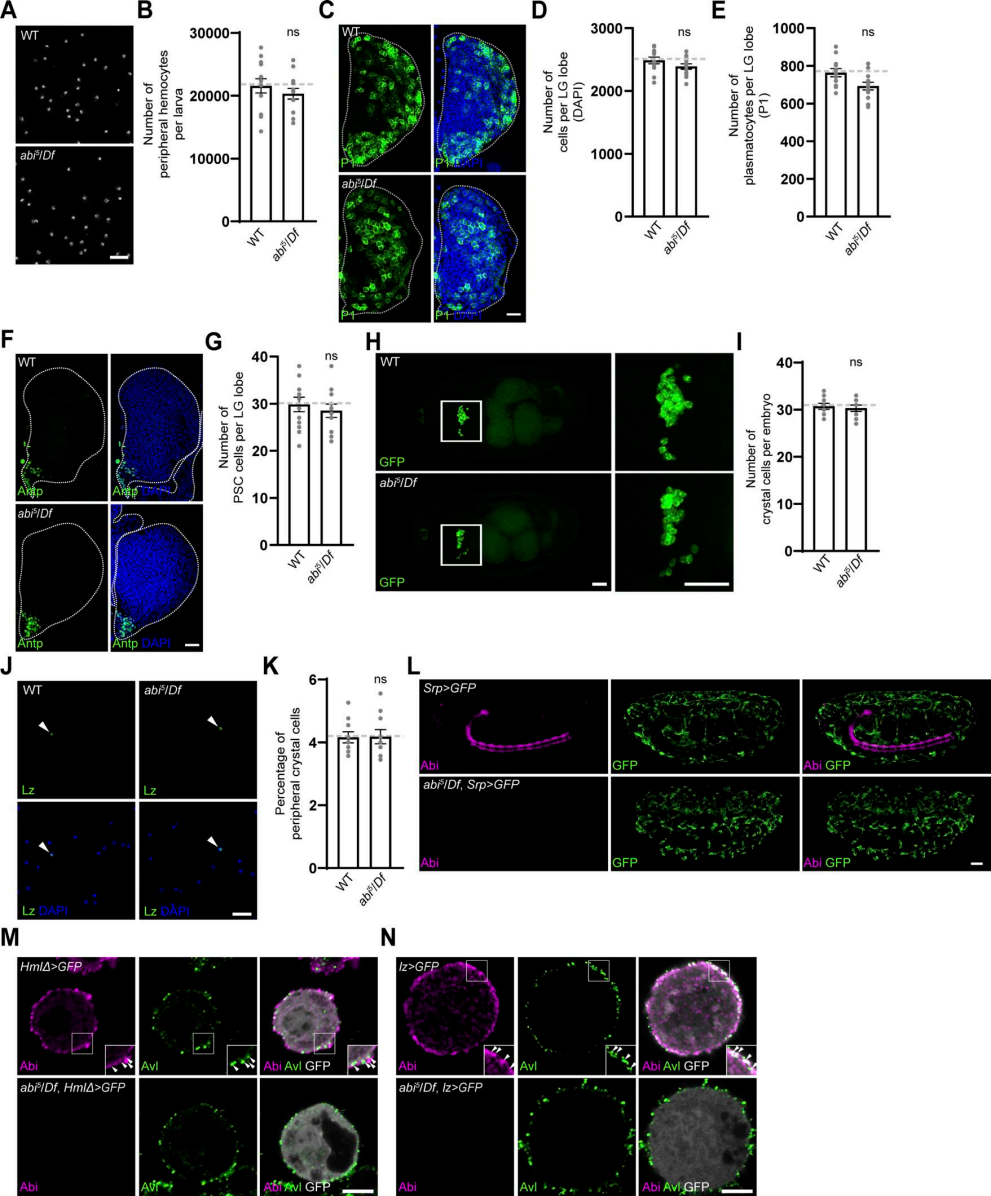
**Hemocyte counts in WT and *abi*** ^***5***^ ***/Df* embryos, larval peripheral hemocyte compartments, and LGs, and analysis of Abi subcellular localization. (A)** Single confocal sections of peripheral hemocytes from WT and *abi*^*5*^*/Df* late-third instar larvae. Total peripheral hemocytes were isolated after vortexing larvae with glass beads and stained with DAPI. **(B)** Number of peripheral hemocytes per larva. *n* = 12 larvae. **(C)** Confocal images of primary LG lobes from WT and *abi*^*5*^*/Df* late-third instar larvae stained with anti-P1 (green) and DAPI (blue). **(D and E)** Numbers of total DAPI-labeled cells (D) and P1^+^ plasmatocytes (E) per primary LG lobe. *n* = 12 lobes. **(F)** Confocal images of primary LG lobes from WT and *abi*^*5*^*/Df* late-third instar larvae stained with anti-Antp (green) and DAPI (blue). **(G)** Numbers of Antp^+^ posterior signaling center cells in primary LG lobes of WT and *abi*^*5*^*/Df* late-third instar larvae. *n* = 10 lobes. **(H)** Confocal images of whole-mount WT and *abi*^*5*^*/Df* embryos (stage 17) carrying *lz-GAL4* and *UAS-mCD8-GFP* (dorsal views). Higher magnification views of boxed areas are shown on the right to highlight GFP^+^ crystal cells clustered around the embryonic proventriculus. **(I)** Number of GFP^+^ crystal cells per embryo. *n* = 10 embryos. **(J)** Confocal images of peripheral hemocytes from WT and *abi*^*5*^*/Df* second instar larvae, stained with an anti-Lz antibody (green) and DAPI (blue). Arrowheads indicate Lz^+^ crystal cells. **(K)** Percentage of Lz^+^ crystal cells among all peripheral hemocytes (total DAPI count). *n* = 10 larvae. **(L)** Confocal images of whole-mount WT and *abi*^*5*^*/Df* embryos at stage 17 (lateral view) carrying *Srp-GAL4* and *UAS-mCD8-GFP*, stained with anti-Abi (pseudocolored magenta) and anti-GFP (green) antibodies. **(M and N)** Confocal images of WT and *abi*^*5*^*/Df* primary hemocytes carrying *HmlΔ-GAL4* and *UAS-EGFP* (M) *or lz-GAL4* and *UAS-mCD8-GFP* (N), stained with anti-Abi (pseudocolored magenta) and anti-Avl (green) antibodies. Insets show high-magnification views in the cell periphery. Arrowheads indicate Abi^+^ puncta colocalizing with Avl. Data represent the mean ± SEM. Student’s *t* test revealed no statistically significant difference (ns). Scale bars: 20 μm (A, C, F, and J); 50 μm (H and L); 5 μm (M and N).

**Figure 1.**
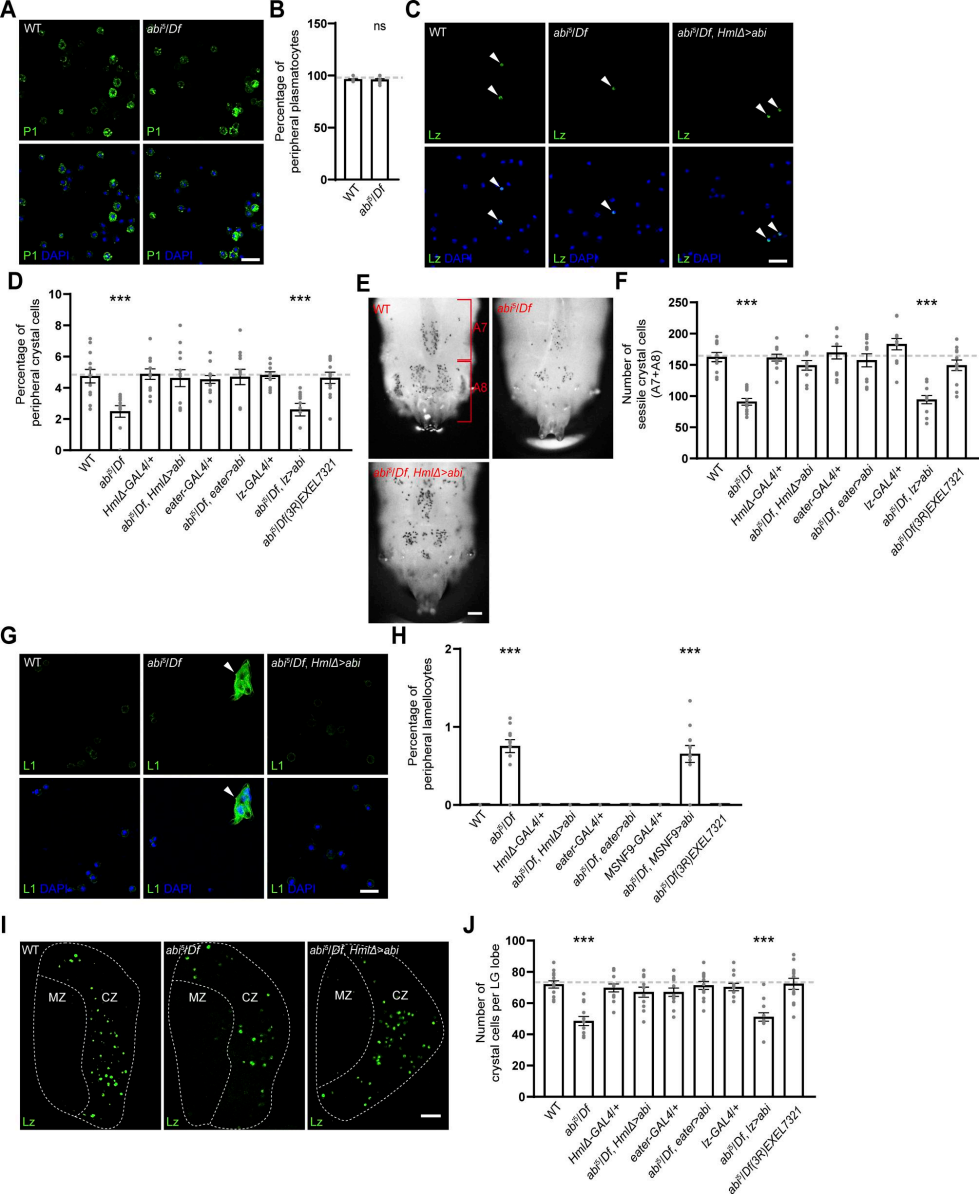
**Abi is required for maintenance of crystal cell and lamellocyte homeostasis during larval development. (A)** Single confocal slices of peripheral hemocytes from WT and *abi*^*5*^*/Df(3R)su(Hw)7* (*abi*^*5*^*/Df*) late-third instar larvae stained with an anti-P1 antibody (green) and DAPI (blue). **(B)** Percentage of P1^+^ plasmatocytes among all peripheral hemocytes (total DAPI count). **(C)** Confocal images of peripheral hemocytes from WT, *abi*^*5*^*/Df*, and *HmlΔ-GAL4*,*abi*^*5*^/*UAS-HA-abi*,*Df* (*abi*^*5*^*/Df*, *HmlΔ > abi*) late-third instar larvae stained with an anti-Lz antibody (green) and DAPI (blue). Arrowheads indicate Lz^+^ crystal cells. **(D)** Percentage of Lz^+^ crystal cells among all peripheral hemocytes (total DAPI count). *abi*^*5*^*/Df*; *eater* > *abi* and *abi*^*5*^*/Df*; *lz* > *abi* represent *eater*-*GAL4*,*abi*^*5*^/*UAS-HA-abi*,*Df* and *lz*-*GAL4*/+; *UAS-HA-abi*,*Df*/*abi*^*5*^, respectively. **(E)** Bright-field images of heated (70°C, 10 min) WT, *abi*^*5*^*/Df*, and *abi*^*5*^*/Df*, *HmlΔ > abi* late-third instar larvae. Dorsal views of the two most posterior segments (A7 and A8). **(F)** Numbers of heat-blackened crystal cells in the A7 and A8 segments of the indicated genotypes. **(G)** Confocal images of peripheral hemocytes from WT, *abi*^*5*^*/Df*, and *abi*^*5*^*/Df*, *HmlΔ > abi* late-third instar larvae stained with an anti-L1 antibody (green) and DAPI (blue). Arrowheads indicate L1^+^ lamellocytes. **(H)** Percentage of L1^+^ lamellocytes among all peripheral hemocytes (total DAPI count). *abi*^*5*^*/Df*, *MSNF9 > abi* represents *MSNF9*-*GAL4*/+; *UAS-HA-abi*,*Df*/*abi*^*5*^. **(I)** Confocal images of primary LG lobes from WT, *abi*^*5*^*/Df*, and *abi*^*5*^*/Df*, *HmlΔ > abi* late-third instar larvae stained with an anti-Lz antibody (green). Optical sections through the middle and two adjacent planes were projected. The dashed lines outline the MZ and CZ of the LG. **(J)** Numbers of Lz^+^ crystal cells in the primary LG lobes for the indicated genotypes. Data represent the mean ± SEM. *n* = 12 larvae/LG lobes. Statistical analyses were performed using Student’s *t* test (B) or a one-way ANOVA with the Tukey–Kramer post hoc test (D, F, H, and J). Comparisons are with WT (***P < 0.001; ns, not significant). Scale bars: 20 μm (A, C, G, and I); 200 μm (E). MZ, medullary zone; CZ, cortical zone.

To investigate the role of Abi in hematopoiesis, we counted peripheral (i.e., circulating plus sessile) hemocytes in third instar larvae transheterozygous for *abi*^*5*^ (an *abi*-null allele) and *Df*(*3R*)*su*(*Hw*)7 (a deficiency deleting the entire *abi* gene; hereafter *Df*) (Kim et al., 2019). The total number of peripheral hemocytes did not differ significantly between wild-type (WT, *w*^*1118*^) and *abi*^*5*^/*Df* larvae (Fig. S1, A and B). Similarly, the abundance of P1/NimC1^+^ plasmatocytes (Kurucz et al., 2007a) was unchanged (Fig. 1, A and B). In contrast, *abi*^*5*^/*Df* larvae displayed significant changes in crystal cells and lamellocyte populations. The frequency of Lozenge^+^ (Lz^+^) crystal cells (Lebestky et al., 2000) was reduced by ∼47%, and the number of sessile crystal cells, visualized by heat-induced melanization, decreased by ∼44% (Fig. 1, C–F). Conversely, the frequency of L1^+^ lamellocytes (Kurucz et al., 2007b) was increased from <0.01% in WT larvae to ∼0.75% in *abi*^*5*^/*Df* larvae (Fig. 1, G and H). No alterations in the abundance of crystal cells and lamellocytes were detected in larvae transheterozygous for *abi*^*5*^ and a smaller deficiency (*Df[3R]EXEL7321*) spanning the *Df*(*3R*)*su*(*Hw*)7 genomic interval but retaining *abi* (Fig. 1, D, F, and H). Thus, loss of Abi oppositely affects the crystal cell and lamellocyte populations in the larval peripheral hematopoietic compartment.

The overall morphology and total hemocyte number of *abi*^*5*^/*Df* primary LG lobes were comparable to WT, as determined by DAPI staining (Fig. S1, C and D). Likewise, the numbers of P1^+^ plasmatocytes and Antennapedia^+^ (Antp^+^) posterior signaling center cells were unchanged (Fig. S1, E–G). In contrast, Lz^+^ crystal cells were selectively reduced by ∼33% in *abi* mutant LGs compared with WT or *abi*^*5*^/*Df(3R)EXEL7321* controls (Fig. 1, I and J), indicating that Abi is also required for crystal cell formation in the LG.

Interestingly, the peripheral crystal cell population in *abi*^*5*^/*Df* mutants was normal until the second instar larval stage (Fig. S1, H–K), a developmental period during which the number of crystal cells remains low (Lanot et al., 2001), suggesting that Abi acts in a developmental stage-specific manner. Consistently, the expression of HA-tagged Abi (HA-Abi) using *HmlΔ-GAL4*, which is active from early larval stages in differentiating precursor cells and plasmatocytes (Charroux and Royet, 2009; Mukherjee et al., 2011), fully rescued the hematopoietic defects in *abi*^*5*^/*Df* larvae (Fig. 1, C–H). To further define the cell type–specific requirement for Abi, we performed rescue experiments using additional hemocyte GAL4 drivers. The expression of HA-Abi in *abi*^*5*^/*Df* mutants under plasmatocyte-specific *eater-GAL4* (Tokusumi et al., 2009) fully restored the abundance of peripheral crystal cells and lamellocytes to WT levels, whereas expression under crystal cell–specific *lz-GAL4* (Lebestky et al., 2000) or lamellocyte-specific *MSNF9-GAL4* (Tokusumi et al., 2009) failed to rescue the same defects (Fig. 1, D, F, and H). Similarly, in the LG, the reduced crystal cell phenotype of *abi*^*5*^/*Df* mutants was rescued by the expression of HA-Abi using *HmlΔ-GAL4* or *eater-GAL4* but not *lz-GAL4* (Fig. 1 J). Thus, Abi is specifically required in differentiated plasmatocytes to maintain hemocyte homeostasis in both the peripheral compartment and the LG.

### Abi localizes to the submembrane cortex in larval hemocytes

To examine Abi expression in hemocytes, we used an antibody raised against a region of the Abi protein (Kim et al., 2019). In *Drosophila* embryos, anti-Abi signals were strongly detected in axonal tracts of the central nervous system, as previously reported (Lin et al., 2009), but were absent in hemocytes labeled by *Srp-GAL4*-driven GFP (Waltzer et al., 2003) (Fig. S1 L). In contrast, prominent anti-Abi signals were detected in larval plasmatocytes and crystal cells, marked respectively by *HmlΔ-GAL4*– and *lz-GAL4*–driven GFP (Fig. S1, M and N). In these cells, Abi was associated predominantly with the plasma membrane, with partial overlap with the early endosome marker Avalanche (Avl).

### The developmental pathway-specific role of Abi in formation of larval and LG crystal cells

Previous studies have shown that larval peripheral crystal cells expand primarily through transdifferentiation of Hml^+^Lz^−^ plasmatocytes into Hml^−^Lz^+^ crystal cells via the intermediate Hml^+^Lz^+^ state (Leitao and Sucena, 2015; Mukherjee et al., 2011). In contrast, crystal cells in the LG arise via two developmental routes: direct differentiation of precursor cells and transdifferentiation from mature plasmatocytes (Banerjee et al., 2019; Marcetteau et al., 2025). The existence of multiple developmental pathways giving rise to mature hemocytes is further supported by single-cell transcriptome analyses (Cattenoz et al., 2020; Cho et al., 2020; Fu et al., 2020; Girard et al., 2021; Tattikota et al., 2020). To better define the developmental origins of larval peripheral crystal cells and investigate the pathway-specific role of Abi, we performed a lineage tracing experiment using the G-TRACE system, in which GAL4 drives the expression of *UAS-RFP* and *UAS-FLP* (Evans et al., 2009). GAL4-expressing cells activate FLP-mediated excision of an FRT-flanked stop cassette, enabling GFP expression from the constitutively active *Ubi-p63E* promoter; thus, real-time GAL4 activity is indicated by RFP and lineage-traced activity by GFP.

**Figure 2.**
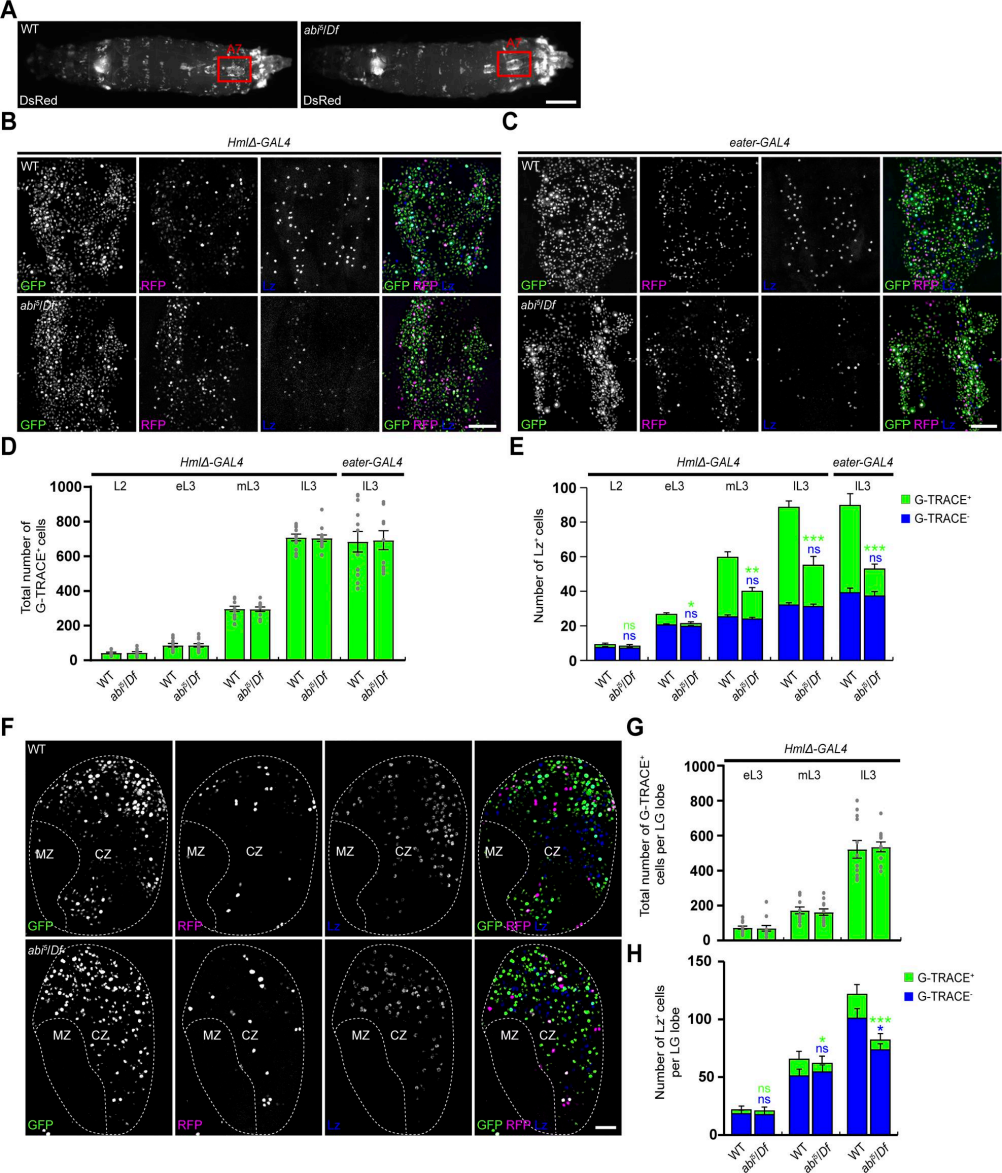
**Loss of Abi reduces the number of *Hml/eater* lineage–traced crystal cells in the peripheral hemocyte compartment and the LG. (A)** Live mount images of WT and *abi*^*5*^/*Df* late-third instar larvae carrying *HmlΔ-DsRed*. The overall distribution of DsRed^+^ hemocytes is normal in *abi*^*5*^/*Df* larvae compared with WT larvae. The red box indicates the dorsal hemocyte cluster in abdominal segment A7. **(B and C)** Confocal images of the dorsal hemocyte cluster in abdominal segment A7 of WT and *abi*^*5*^/*Df* late-third instar larvae carrying *HmlΔ-GAL4* (B) or *eater-GAL4* (C) together with *UAS-G-TRACE* (GFP in green and RFP in pseudocolored magenta), stained with an anti-Lz antibody (blue). **(D)** Numbers of *Hml* and *eater* lineage–traced cells in larvae at 60, 78, 92, and 112 h AEL at 25°C, corresponding to the second instar (L2), and early (e)-, mid (m)-, and late (l)-third instar (L3) stages, respectively. G-TRACE–labeled (G-TRACE^+^) cells were quantified as the total number of GFP^+^ only (green), RFP^+^ plus GFP^+^ (yellow), and RFP^+^-only (red) cells. **(E)** Numbers of Lz^+^ crystal cells at the indicated larval stages. Lz^+^ crystal cells were counted and classified as G-TRACE^+^ and G-TRACE^-^. **(F)** Confocal images of primary LG lobes from WT and *abi*^*5*^/*Df* late-third instar larvae carrying *HmlΔ-GAL4* together with *UAS-G-TRACE*, stained with an anti-Lz antibody. The dashed line outlines the LG lobe. **(G)** Numbers of *Hml* lineage–traced cells in primary LG lobes at the indicated larval stages. **(H)** Numbers of Lz^+^ crystal cells in primary LG lobes at the indicated larval stages. Data represent the mean ± SEM. *n* = 12 larvae/LG lobes. Statistical analyses were performed using Student’s *t* test. Comparisons are with WT (*P < 0.05; **P < 0.01; ***P < 0.001; ns, not significant). Scale bars: 500 μm (A); 50 μm (B and C); 20 μm (F).

We activated G-TRACE with plasmatocyte-specific *HmlΔ-GAL4* and stained larvae for Lz at second (60 h after egg laying [AEL]), early-third (78 h AEL), mid-third (96 h AEL), and late-third (112 h AEL) instar stages. After confirming that peripheral distribution of Hml^+^ plasmatocytes was normal in *abi*^*5*^/*Df* larvae (Fig. 2 A), we analyzed the major sessile hemocyte cluster on the dorsal side of abdominal segment A7. In WT larvae, *Hml* lineage–traced plasmatocytes (GFP^+^ and/or RFP^+^ cells) remained sparse until the second instar stage, but rapidly expanded during the third instar stage (Fig. 2, B and D). Similar to Hml^+^ hemocytes, Lz^+^ crystal cells also increased mainly during the third instar stage (Fig. 2, B and E). Interestingly, ∼64% of Lz^+^ cells were *Hml* lineage–traced, whereas ∼36% were unmarked (Fig. 2 E), revealing alternative developmental pathways for crystal cell formation. Loss of Abi selectively impaired expansion of *Hml* lineage–traced Lz^+^ cells without affecting non-lineage–traced Lz^+^ cells (Fig. 2 E), revealing a specific role in promoting transdifferentiation of Hml^+^ plasmatocytes into Lz^+^ crystal cells. This conclusion was reinforced using plasmatocyte-specific *eater-GAL4*, which labeled ∼55% of Lz^+^ cells (Fig. 2, C and E). Loss of Abi specifically reduced *eater* lineage–derived crystal cells but not non-lineage–traced cells (Fig. 2, C and E). As a control, *lz-GAL4*–driven G-TRACE labeling marked ∼95% of all Lz^+^ cells (data not shown), confirming that the observed ∼36% of *Hml* non-lineage–traced Lz^+^ cells reflects true developmental heterogeneity rather than a tracing limitation.

**Figure S2.**
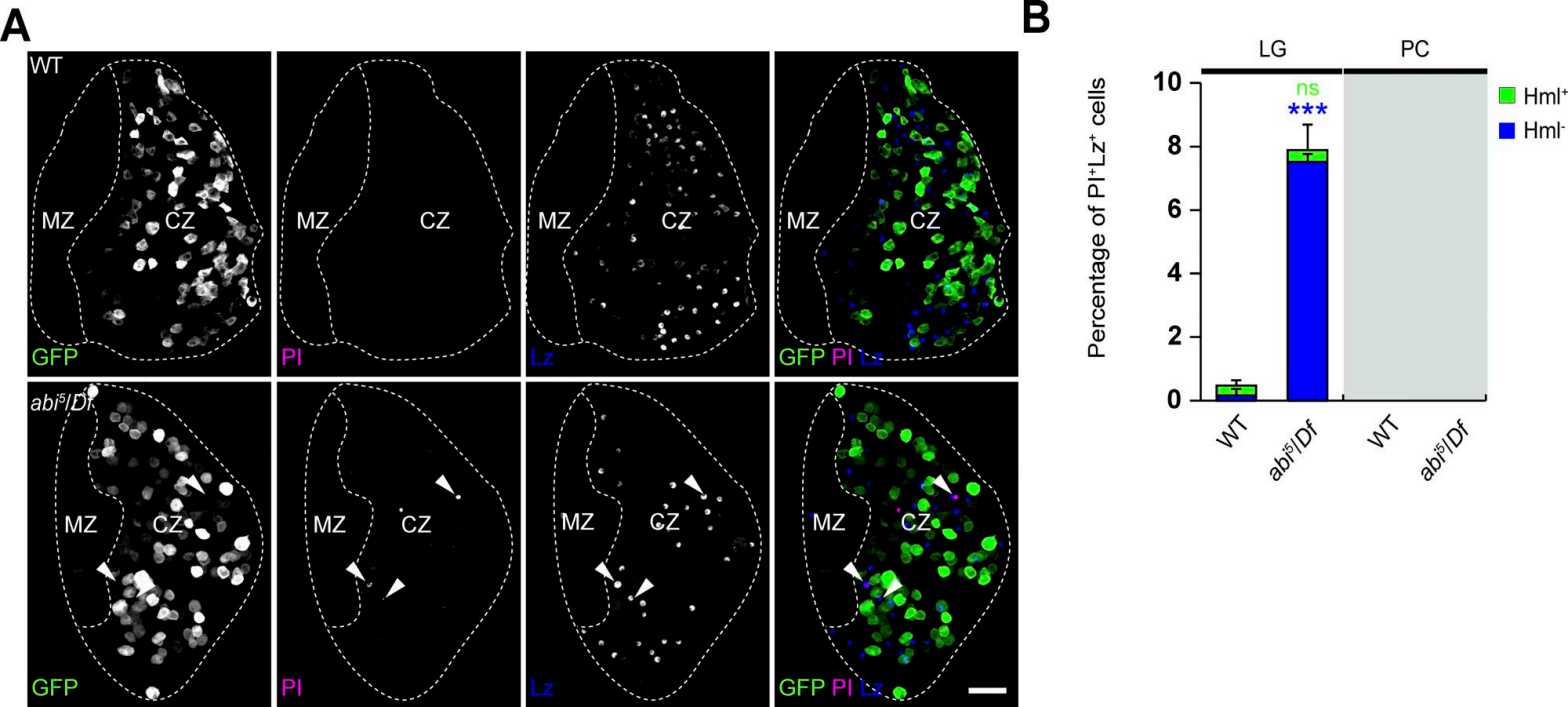
**Analysis of cell death in *abi* mutant hemocytes. (A)** Confocal images of primary LG lobes from WT and *abi*^*5*^/*Df* late-third instar larvae carrying *HmlΔ-GAL4* together with *UAS-EGFP* (green), stained with an anti-Lz antibody (blue) and PI (pseudocolored magenta). Arrowheads indicate Lz^+^PI^+^ crystal cells. **(B)** Percentage of Lz^+^PI^+^ cells among total Lz^+^ crystal cells in the LG and peripheral hematopoietic compartment (PC). Lz^+^PI^+^ cells were classified as Hml^+^ (green) or Hml^−^ (blue). *n* = 9 lobes/larvae. Data represent the mean ± SEM. Statistical analyses were performed using Student’s *t* test. Comparisons are with WT (***P < 0.001; ns, not significant). Scale bar: 20 μm.

We next investigated the lineage-specific role of Abi in the LG. As in the periphery, *Hml* lineage plasmatocytes and Lz^+^ crystal cells expanded primarily during the late-third instar stage (Fig. 2, F and G). However, *Hml* lineage–traced Lz^+^ cells represented a much smaller fraction of total Lz^+^ crystal cells (∼18% at late-third instar; Fig. 2 H), indicating that transdifferentiation of Hml^+^ plasmatocytes contributes only modestly to LG crystal cell expansion. From the mid-third instar onward, *Hml* lineage–traced Lz^+^ cells were reduced in *abi*^*5*^/*Df* larvae relative to WT (Fig. 2 H), supporting a role of Abi in promoting plasmatocyte-to-crystal cell transdifferentiation within the LG. Notably, loss of Abi did not affect non-lineage–traced Lz^+^ cells until mid-third instar but caused a significant reduction by late-third instar (Fig. 2 H), consistent with an additional role of Abi in supporting LG crystal cell survival, as confirmed by a propidium iodide (PI) exclusion (Fig. S2).

### Abi promotes plasmatocyte-to-crystal cell transdifferentiation by activating Notch signaling

**Figure 3.**
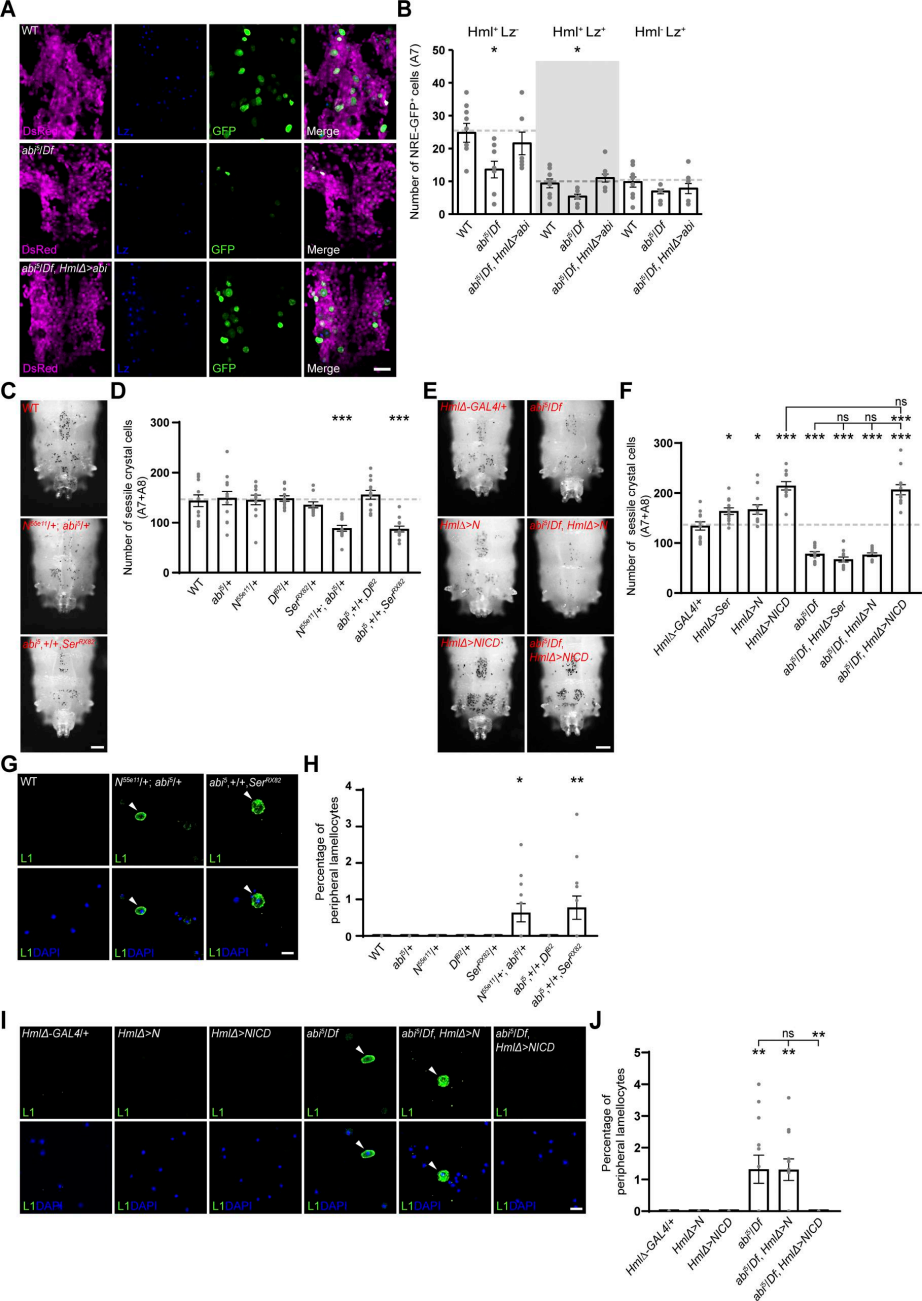
**Abi regulates hematopoietic homeostasis by activating Notch signaling. (A and B)** Abi is required for activation of Notch signaling in hemocytes of the Hml lineage. **(A)** Confocal images of dorsal hemocyte clusters (abdominal segment A7) in WT, *abi*^*5*^/*Df*, and *HmlΔ*-*GAL4*,*abi*^*5*^/*UAS-HA-abi*,*Df* (*abi*^*5*^*/Df*, *HmlΔ > abi*) late-third instar larvae carrying *HmlΔ*-*DsRed* (pseudocolored magenta) and the Notch signaling reporter *NRE-GFP* (green). Larvae were stained with an anti-Lz antibody (blue). **(B)** Numbers of NRE-GFP^+^ hemocytes counted and classified as Hml^+^Lz^−^, Hml^+^Lz^+^, or Hml^−^Lz^+^. **(C and D)** Transheterozygous interaction between *abi* and *Ser* or *Notch* (*N*). **(C)** Bright-field images of WT, *N*^*55e11*^/+; *abi*^*5*^/+, and *abi*^*5*^,+/+,*Ser*^*RX82*^ late-third instar larvae (heated at 70°C for 10 min). Dorsal views of the two most posterior segments (A7 and A8). **(D)** Numbers of heat-blackened crystal cells in the A7 and A8 segments of the indicated genotypes. **(E and F)** Mutations in *abi* suppress the excess crystal cell phenotype caused by the overexpression of full-length *N*, but not *NICD*. **(E)** Bright-field images of *HmlΔ-GAL4*/+, *HmlΔ-GAL4*/*UAS-N* (*HmlΔ* > *N*), *UAS-NICD*/+; *HmlΔ-GAL4*/+ (*HmlΔ* > *NICD*), *abi*^*5*^*/Df*, *HmlΔ-GAL4*,*abi*^*5*^/*UAS-N*,*Df* (*abi*^*5*^*/Df*, *HmlΔ* > *N*), and *UAS-NICD*/+; *HmlΔ-GAL4*,*abi*^*5*^/+,*Df* (*abi*^*5*^*/Df*, *HmlΔ* > *NICD*) late-third instar larvae (heated at 70°C for 10 min). Dorsal views of the two most posterior segments (A7 and A8). **(F)** Numbers of heat-blackened crystal cells in segments A7 and A8. The genotypes analyzed additionally include *HmlΔ-GAL4*/*UAS-Ser* (*HmlΔ > Ser*) and *HmlΔ-GAL4*,*abi*^*5*^/*UAS-Ser*,*Df* (*abi*^*5*^*/Df*, *HmlΔ > Ser*). **(G)** Confocal images of peripheral hemocytes from WT, *N*^*55e11*^/+; *abi*^*5*^/+, and *abi*^*5*^,+/+,*Ser*^*RX82*^ late-third instar larvae stained with an anti-L1 antibody (green) and DAPI (blue). Arrowheads indicate L1^+^ lamellocytes. **(H)** Percentage of L1^+^ lamellocytes among all peripheral hemocytes (total DAPI count). **(I and J)** Overexpression of NICD, but not full-length Notch, completely suppresses the excess lamellocyte phenotype caused by *abi* mutations. **(I)** Confocal images of peripheral hemocytes from late-third instar larvae of the indicated genotypes stained with an anti-L1 antibody (green) and DAPI (blue). Arrowheads indicate L1^+^ lamellocytes. **(J)** Percentage of L1^+^ lamellocytes among all peripheral hemocytes (total DAPI count). Data represent the mean ± SEM. *n* = 12 larvae. Statistical analyses were performed using a one-way ANOVA with the Tukey–Kramer post hoc test. Comparisons are with WT or *HmlΔ-GAL4*/+ unless otherwise indicated (*P < 0.05; **P < 0.01; ***P < 0.001; ns, not significant). Scale bars: 20 μm (A, G, and I); 200 μm (C and E).

Given the role of Ser-dependent Notch signaling in transdifferentiation of Hml^+^Lz^−^ plasmatocytes into Hml^−^Lz^+^ crystal cells (Leitao and Sucena, 2015; Marcetteau et al., 2025), we hypothesized that Abi might act by activating Notch signaling. To test this hypothesis, we focused our analysis on the peripheral hematopoietic compartment, where plasmatocyte transdifferentiation occurs more prevalently than in the LG. We first examined Notch activity in sessile hemocyte clusters in abdominal segment A7 of larvae expressing the hemocyte reporter *HmlΔ-DsRed* (Makhijani et al., 2011) and the Notch reporter *NRE-GFP* (Saj et al., 2010), stained with anti-Lz. In the WT background, GFP was detected in subsets of Hml^+^Lz^−^, Hml^+^Lz^+^, and Hml^−^Lz^+^ cells (Fig. 3, A and B). Interestingly, loss of Abi selectively reduced GFP^+^ cells within the Hml^+^Lz^−^ and Hml^+^Lz^+^ populations but not within Hml^−^Lz^+^ cells (Fig. 3, A and B). This reduction was fully rescued by *HmlΔ-GAL4–*driven *UAS-HA-abi* expression, supporting that Abi acts in Hml^+^ hemocytes to activate Notch signaling during crystal cell formation.

We next tested whether *abi* genetically interacts with *Notch* (*N*) and its ligands, *Ser* and *Delta* (*Dl*), using strong loss-of-function alleles (*N*^*55e11*^, *Ser*^*RX82*^, and *Dl*^*B2*^) (Micchelli et al., 1997; Rulifson and Blair, 1995; Thomas et al., 1991). Heterozygous *abi*^*5*^/+, *N*^*55e11*^/+, *Ser*^*RX82*^/+, and *Dl*^*B2*^/+ larvae showed normal numbers of sessile crystal cells, as did *abi*^*5*^,+/+,*Dl*^*B2*^ transheterozygotes. However, the number of sessile crystal cells was greatly reduced in transheterozygous *N*^*55e11*^/+; *abi*^*5*^/+ and *abi*^*5*^,+/+,*Ser*^*RX82*^ larvae (Fig. 3, C and D), supporting a functional connection between Abi and Ser/Notch signaling during crystal cell formation.

This conclusion was further tested by performing epistasis analysis. As reported (Duvic et al., 2002; Mukherjee et al., 2011), the overexpression of full-length Notch (*UAS-N*) or Ser (*UAS-Ser*) with *HmlΔ-GAL4* increased sessile crystal cell numbers (Fig. 3, E and F). This Notch gain-of-function phenotype was fully suppressed by loss of Abi, as Notch-overexpressing *abi*^*5*^/*Df* mutants displayed crystal cell numbers indistinguishable from naïve *abi*^*5*^/*Df* mutants (Fig. 3, E and F), demonstrating that Notch requires Abi to promote hematopoiesis. The overexpression of Notch intracellular domain (NICD), which is the product of γ-secretase–mediated Notch activation (Struhl and Adachi, 1998), induced an even stronger increase in crystal cells (Fig. 3, E and F). Notably, this phenotype was not suppressed by loss of Abi. Together, these data support a model in which Abi promotes crystal cell formation by activating Notch signaling upstream of NICD release.

### Abi-mediated activation of Notch signaling is also required for repression of lamellocyte differentiation

In contrast to promoting plasmatocyte-to-crystal cell transdifferentiation, Notch signaling represses plasmatocyte-to-lamellocyte transdifferentiation in healthy larvae (Small et al., 2014). We therefore asked whether the excess lamellocyte phenotype of *abi*^*5*^/*Df* larvae results from impaired Notch signaling. The percentage of lamellocytes among total peripheral hemocytes was similar to WT in heterozygous *abi*^*5*^/+, *N*^*55e11*^/+, *Ser*^*RX82*^/+, and *Dl*^*B2*^/+ larvae (Fig. 3, G and H). However, lamellocyte percentages were greatly increased in *N*^*55e11*^/+; *abi*^*5*^/+ and *abi*^*5*^,+/+,*Ser*^*RX82*^ larvae but not in *abi*^*5*^,+/+,*Dl*^*B2*^ larvae (Fig. 3, G and H), supporting the idea that Abi represses lamellocyte formation through Ser/Notch signaling.

Epistasis analysis further confirmed this conclusion. The *HmlΔ-GAL4*–driven overexpression of full-length Notch or NICD did not affect lamellocyte differentiation in WT larvae (Fig. 3, I and J). Importantly, the overexpression of NICD, but not full-length Notch, completely suppressed the excess lamellocyte phenotype in *abi*^*5*^/*Df* mutants (Fig. 3, I and J). These results indicate that Abi represses transdifferentiation of Hml^+^ plasmatocytes into lamellocytes by activating Notch signaling.

### Abi activates Notch signaling by mediating receptor internalization via CME

**Figure 4.**
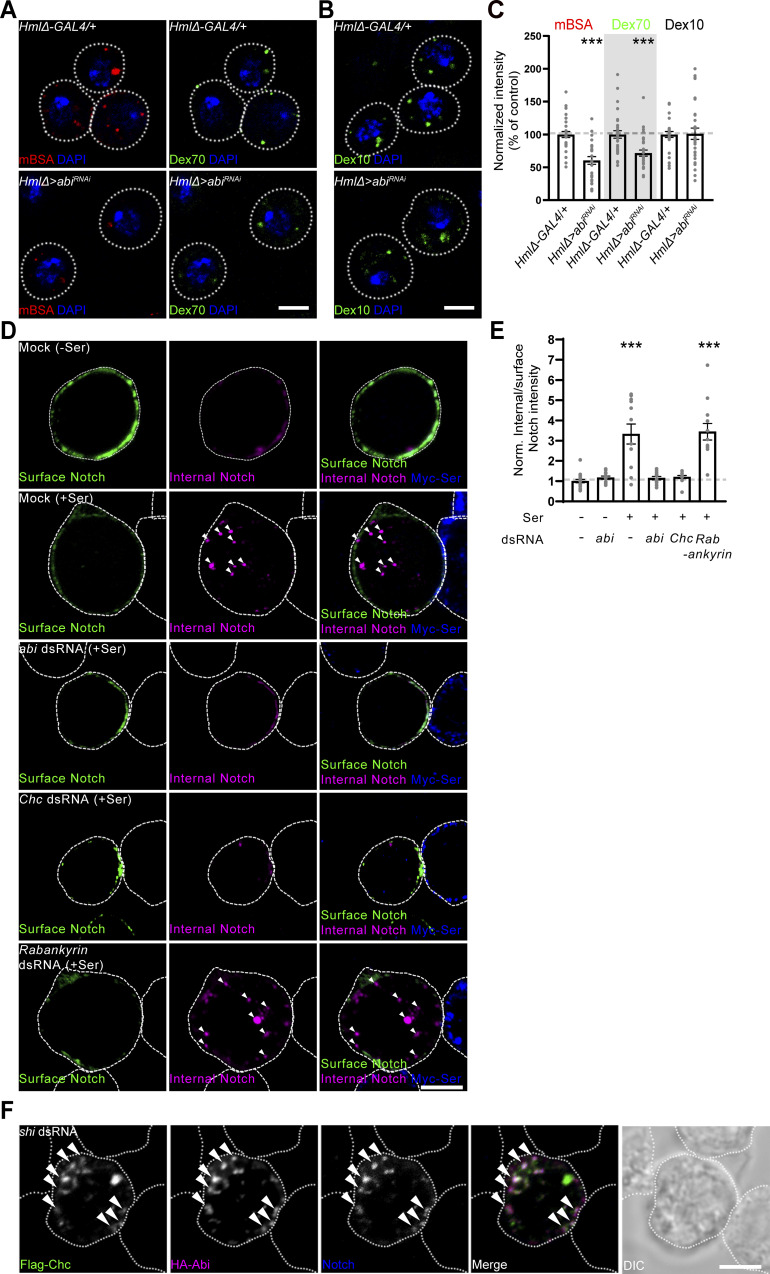
**Abi-dependent CME is required for ligand-induced Notch internalization. (A and B)** Single confocal slices of primary hemocytes from *HmlΔ-GAL4*/+ and *UAS-abi*^*RNAi*^/+; *HmlΔ*-*GAL4*/+ (*HmlΔ* > *abi*^*RNAi*^) late-third instar larvae. Hemocytes were pulsed with Alexa Fluor 555-mBSA (mBSA, a CME marker) and 70 kDa FITC-dextran (Dex70, a macropinocytosis marker) (A) or 10 kDa FITC-dextran (Dex10, a GEEC endocytosis marker) alone (B) for 5 min, chased for 5 min, and stained with DAPI. **(C)** Quantification of endocytic events in A and B. The ratios of mBSA, Dex70, and Dex10 to DAPI fluorescence intensities are presented as percentages of *HmlΔ-GAL4*/+. *n* = 30 hemocytes. **(D)** Single confocal slices of S2N cells transfected with *abi*, *Chc*, or *Rabankyrin* dsRNA. Transfected S2N cells were pretreated with 0.7 mM CuSO_4_ and cocultured with S2S cells. Live cocultured cells were incubated with an anti-NECD antibody at 4°C for 30 min to prelabel surface Notch receptors (on S2N cells) and further incubated at 25°C for 30 min to allow endocytosis to resume. After fixation, cocultured cells were sequentially stained for surface (green) and internalized (pseudocolored magenta) Notch receptors under nonpermeant and permeant conditions, respectively. Permeabilized cocultured cells were additionally stained for Myc-Ser (blue). Arrowheads indicate intracellular punctate structures containing internalized Notch. **(E)** Quantification of the ratio of internal to surface Notch fluorescence intensities. *n* = 9 cells. **(F)** Single confocal slices of S2N cells transfected with *shi* dsRNA, pretreated with 0.7 mM CuSO_4_ for 24 h, and cocultured with S2S cells for 6 h. Notch receptors on S2N cells were prelabeled with an anti-NECD antibody and allowed to internalize as in D. After fixation and permeabilization, cells were stained for Flag-Chc (green), HA-Abi (pseudocolored magenta), and prelabeled Notch (blue). Arrowheads indicate colocalization of Flag-Chc, HA-Abi, and Notch. Data represent the mean ± SEM. Statistical analyses were performed using Student’s *t* test (C) or by a one-way ANOVA with the Tukey–Kramer post hoc test (E). Comparisons are with *HmlΔ-GAL4*/+ in C and mock-transfected isolated (-Ser) S2N cells in E (***P < 0.001). Scale bars: 5 μm (A and B); 10 μm (D and F).

CME is required for activation of ligand-induced Notch signaling (Chapman et al., 2016; Windler and Bilder, 2010). This requirement is supported by the finding that γ-secretase–mediated Notch cleavage occurs efficiently in the endosomal system (Chastagner et al., 2017; Gupta-Rossi et al., 2004; Vaccari et al., 2008). Given Abi’s role in actin filament assembly, which is essential for endocytosis (Mooren et al., 2012), and its localization to the plasma membrane and Avl^+^ endosomes, Abi may promote Notch signaling by facilitating receptor endocytosis. Before testing this hypothesis, we analyzed the role of Abi in different endocytic pathways in primary hemocytes using maleylated bovine serum albumin (mBSA, a CME probe) (Guha et al., 2003), fluorescein isothiocyanate (FITC)–labeled 10-kDa dextran (Dex10, a GPI-enriched endocytic compartment endocytosis probe) (Kim et al., 2017), and FITC-labeled 70-kDa dextran (Dex70, a macropinocytosis probe) (Kim et al., 2019). Primary hemocytes from control larvae could uptake all of these endocytic probes (Fig. 4, A–C). Knockdown of Abi significantly reduced uptake of mBSA or Dex70 but not Dex10 (Fig. 4, A–C), demonstrating that Abi is specifically required for CME and macropinocytosis. Based on this finding, we used multiple approaches to examine the relevance and specificity of Abi-dependent CME and macropinocytosis in regulating Notch internalization and signal transduction.

**Figure S3.**
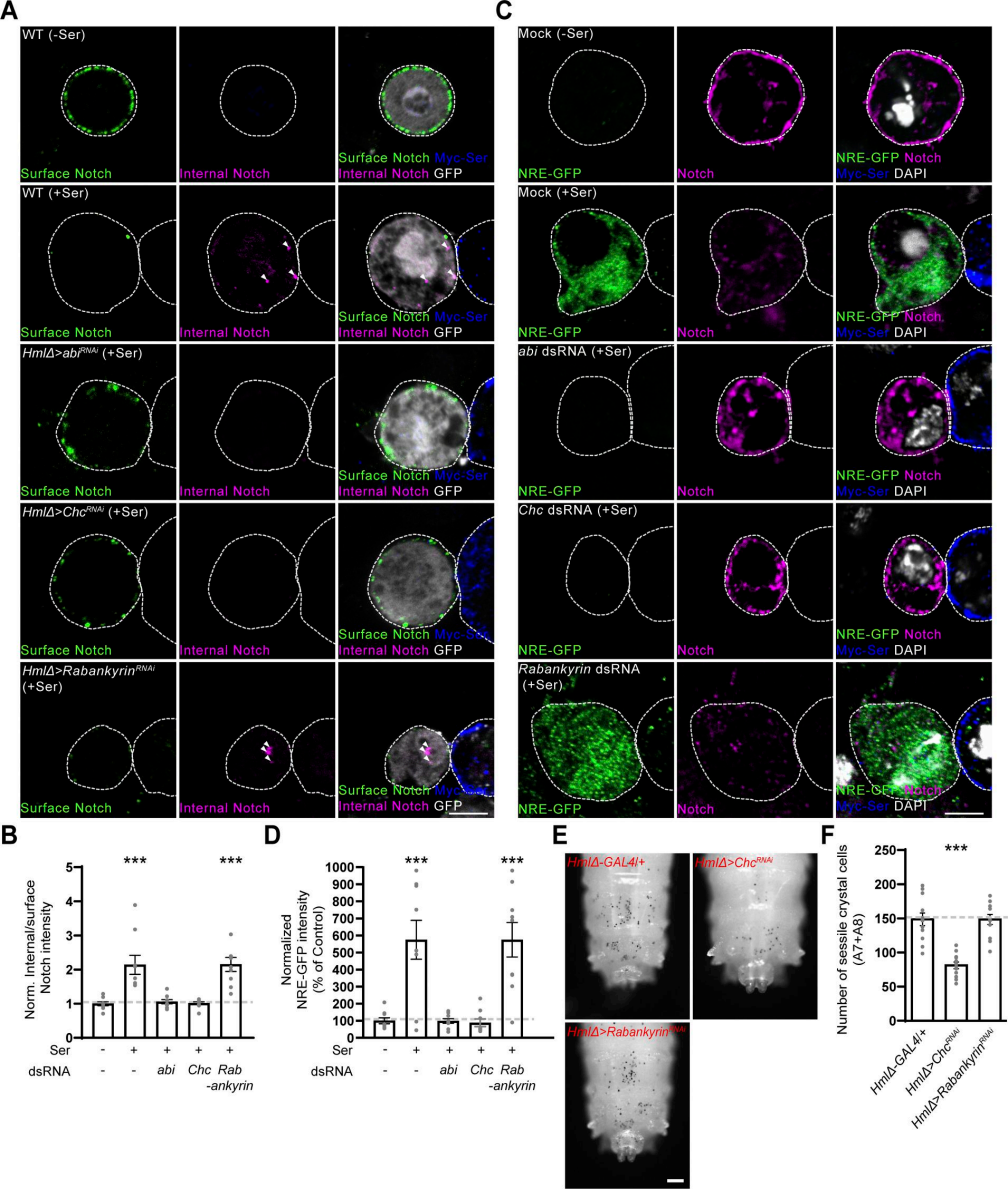
**Abi-dependent CME is required for ligand-induced Notch internalization and signaling activation. (A)** Single confocal slices of primary hemocytes from *HmlΔ-GAL4*,*UAS-EGFP*/+ (WT), *UAS-abi*^*RNAi*^/+; *HmlΔ-GAL4*,*UAS-EGFP*/+ (*HmlΔ* > *abi*^*RNAi*^), *HmlΔ-GAL4*,*UAS-EGFP*/*UAS-Chc*^*RNAi*^ (*HmlΔ* > *Chc*^*RNAi*^), *HmlΔ-GAL4*,*UAS-EGFP*/*UAS-Rabankyrin*^*RNAi*^ (*HmlΔ* > *Rabankyrin*^*RNAi*^) late-third instar larvae. Primary hemocytes were cocultured with S2S cells, and subjected to a Notch internalization assay as in Fig. 4 D. Arrowheads indicate intracellular punctate structures containing internalized Notch. **(B)** Quantification of the ratio of internal to surface Notch fluorescence intensities. *n* = 9 hemocytes. **(C)** Single confocal slices of S2N cells transfected with *NRE-GFP* with or without *abi*, *Chc*, or *Rabankyrin* dsRNA and cocultured with S2S cells for 6 h, prior to immunofluorescence analysis using anti-GFP (green), anti-NECD (pseudocolored magenta), and anti-Myc (for Myc-Ser on S2S cells; blue) antibodies and DAPI (white). **(D)** Quantification of the ratio of mean GFP to DAPI fluorescence intensities. Values are percentages of mock-transfected isolated (-Ser) cells. *n* = 9 cells. **(E)** Bright-field images of heated (70°C, 10 min) *HmlΔ-GAL4*/+, *HmlΔ-GAL4*/*UAS-Chc*^*RNAi*^ (*HmlΔ > Chc*^*RNAi*^), and *HmlΔ-GAL4*/*UAS-Rabankyrin*^*RNAi*^ (*HmlΔ > Rabankyrin*^*RNAi*^) late-third instar larvae. Dorsal views of the two most posterior segments (A7 and A8). **(F)** Numbers of heat-blackened crystal cells in the A7 and A8 segments. *n* = 12 larvae. Data represent the mean ± SEM. Statistical analyses were performed using a one-way ANOVA with the Tukey–Kramer post hoc test (***P < 0.001). Scale bars: 10 μm (A and C); 200 μm (E).

First, we compared the effects of knocking down Abi, clathrin heavy chain (Chc), or the macropinocytosis regulator Rabankyrin on Ser-induced Notch internalization. To this end, we established a Notch internalization assay using hemocyte-derived *Drosophila* S2 cells stably expressing Notch (S2N cells) cocultured with Myc-Ser–expressing S2 cells (S2S cells). In this assay, surface Notch receptors in live S2N cells were prelabeled at 4°C with an antibody against the Notch extracellular domain (NECD). After a 30-min chase at room temperature, surface and internalized Notch receptors were sequentially monitored by antibody staining before and after cell permeabilization, respectively. Isolated S2N cells had a low internal-to-surface Notch ratio, which was not changed by Abi knockdown (Fig. 4, D and E). Upon contact with S2S cells, the internal-to-surface Notch ratio of S2N cells significantly increased (∼233%) (Fig. 4, D and E). Importantly, this Ser-induced enhancement was completely blocked by knockdown of Abi or Chc, but not Rabankyrin, in S2N cells (Fig. 4, D and E), indicating that Abi-dependent CME plays a role in Ser-induced Notch internalization. We further validated this notion in primary hemocytes, where the Notch internalization assay likewise revealed critical roles of Abi and Chc in the internalization of endogenous Notch (Fig. S3, A and B). Consistently, upon coculture with S2S cells, S2N cells exhibited strong colocalization of HA-Abi with surface-labeled Notch on Flag-Chc^+^ clathrin-coated pits (CCPs) that were stabilized by knockdown of the dynamin ortholog Shibire (Shi) (Fig. 4 F, arrowheads).

Second, we compared the effects of Abi, Chc, and Rabankyrin knockdown on Notch signaling in S2N cells. The activity of Notch signaling was significantly higher in S2N cells contacting Myc-Ser cells than in isolated S2N cells, as demonstrated by NRE-GFP expression (Fig. S3, C and D). This Ser-induced enhancement was completely blocked by knockdown of Abi or Chc, but not Rabankyrin, in S2N cells (Fig. S3, C and D), suggesting that Abi-dependent CME plays a role in Ser-dependent Notch signaling.

Finally, we compared the effects of Chc and Rabankyrin knockdown on the population of larval sessile crystal cells. Knockdown of Chc using *HmlΔ-GAL4* mimicked the phenotype of *abi*^*5*^/*Df* mutants by significantly reducing the number of crystal cells, while knockdown of Rabankyrin had no effect (Fig. S3, E and F). These findings support the conclusion that Abi-dependent CME, but not Abi-dependent macropinocytosis, is required for Notch signaling activation during crystal cell formation.

### Abi acts together with WASp to promote CME and crystal cell formation

**Figure 5.**
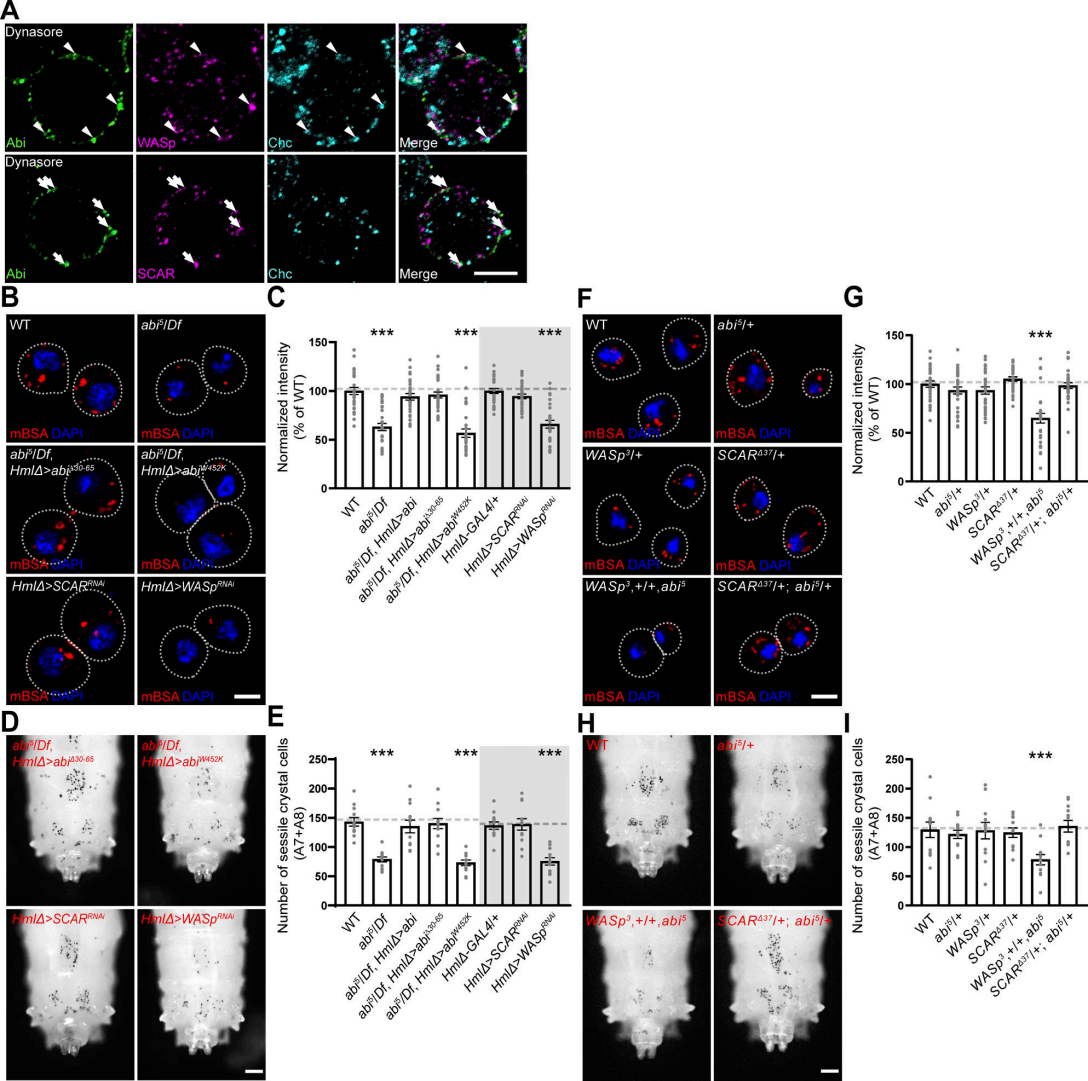
**Abi acts through WASp to regulate CME and crystal cell development. (A)** Single confocal slices of S2 cells pretreated with 100 μM dynasore for 30 min prior to immunofluorescence analysis using anti-Abi (green) and anti-Chc (cyan) antibodies together with an anti-WASp or anti-SCAR (pseudocolored magenta) antibody. Arrowheads indicate Abi^+^ punctae colocalizing with WASp and Chc. Arrows indicate Abi^+^ punctae colocalizing with SCAR but not Chc. **(B)** Single confocal slices of primary hemocytes from WT, *abi*^*5*^*/Df*, *UAS-HA-abi*^*Δ30–65*^/+; *HmlΔ-GAL4*,*abi*^*5*^/+,*Df* (*abi*^*5*^*/Df*, *HmlΔ > abi*^*Δ30–65*^), *HmlΔ-GAL4*,*abi*^*5*^/*UAS-HA-abi*^*W452K*^,*Df* (*abi*^*5*^*/Df*, *HmlΔ > abi*^*W452K*^), *HmlΔ-GAL4*/+, *HmlΔ-GAL4*/*UAS-SCAR*^*RNAi*^ (*HmlΔ > SCAR*^*RNAi*^), and *HmlΔ-GAL4*/*UAS-WASp*^*RNAi*^ (*HmlΔ > WASp*^*RNAi*^) late-third instar larvae. Hemocytes were pulsed with Alexa Fluor 555-mBSA (red) for 5 min, chased for 5 min, and stained with DAPI (blue). **(C)** Quantification of the ratio of mean Alexa Fluor 555-mBSA to DAPI fluorescence intensities. *HmlΔ-GAL4*,*abi*^*5*^/*UAS-HA-abi*,*Df* (*abi*^*5*^*/Df*, *HmlΔ > abi*) was also analyzed. Values are presented as percentages of WT or *HmlΔ-GAL4*/+. *n* = 30 hemocytes. **(D)** Bright-field images of heated (70°C, 10 min) late-third instar larvae of the indicated genotypes. Dorsal views of the two most posterior segments (A7 and A8). **(E)** Number of heat-blackened crystal cells in the A7 and A8 segments. *n* = 12 larvae. **(F–I)** Transheterozygous interactions between *abi* and *WASp*. **(F)** Single confocal slices of primary hemocytes from late-third instar larvae of the indicated genotypes. Hemocytes were pulsed with Alexa Fluor 555-mBSA for 5 min and chased for 5 min. **(G)** Quantification of the ratio of mean Alexa Fluor 555-mBSA to DAPI fluorescence intensities. Values are presented as percentages of WT. *n* = 30 hemocytes. **(H)** Bright-field images of heated (70°C, 10 min) late-third instar larvae of the indicated genotypes. **(I)** Number of heat-blackened crystal cells in the A7 and A8 segments. *n* = 12 larvae. Data represent the mean ± SEM. Comparisons are with WT or *HmlΔ-GAL4*/+ (***P < 0.001). Statistical analyses were performed using a one-way ANOVA with the Tukey–Kramer post hoc test. The dashed lines define cell boundaries. Scale bars: 10 μm (A); 5 μm (B and F); 200 μm (D and H).

Previous work in *Drosophila* showed that Abi and SCAR, but not WASp, are essential for macropinocytosis (Kim et al., 2019). Conversely, Abi binds and activates WASp to promote bristle formation, another actin-based process (Bogdan et al., 2005). To investigate the functional relevance of SCAR and WASp in Abi-dependent CME, we first analyzed their localization to Abi^+^ CCPs at an early stage of CME. When CCPs in S2 cells were stabilized by treatment with the dynamin inhibitor dynasore, anti-WASp signals largely localized to punctate structures that were colabeled for Abi and Chc (Fig. 5 A, arrowheads). However, anti-SCAR signals overlapped with Abi^+^ punctae that were devoid of Chc (Fig. 5 A, arrows). These results suggest that WASp plays a role in Abi-mediated CME.

**Figure S4.**
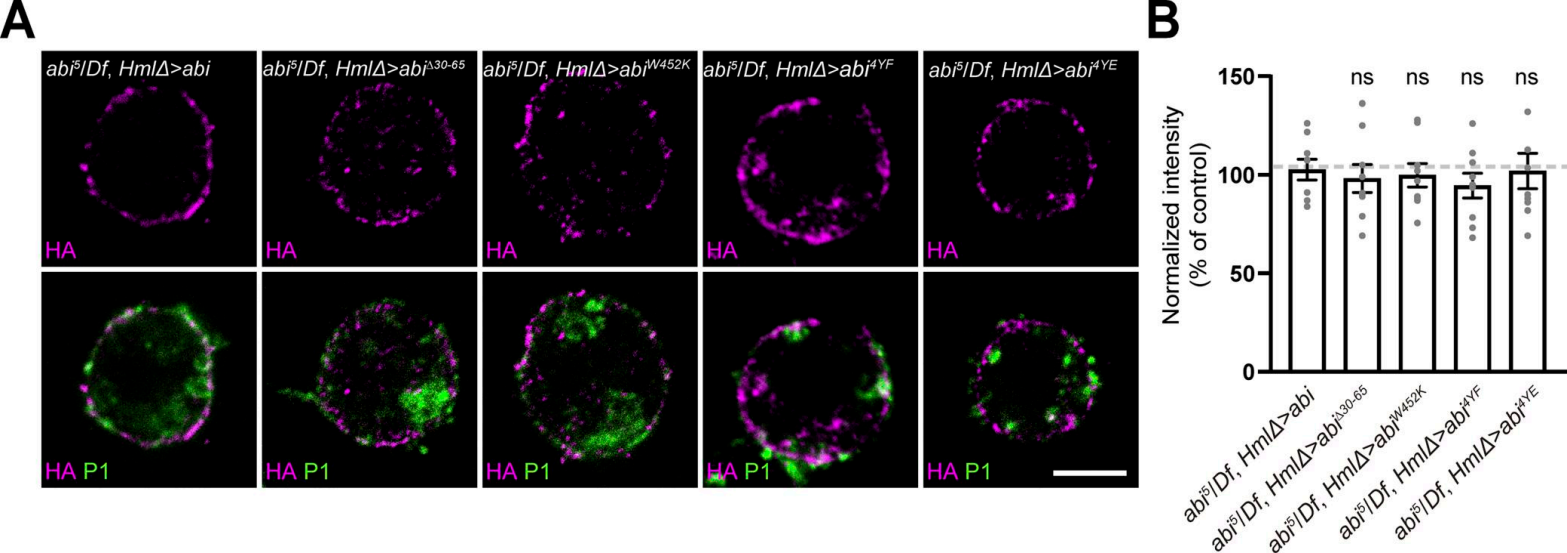
**Analysis of the transgenic expression of HA-Abi and its variants. (A)** Confocal images of primary hemocytes from *HmlΔ-GAL4*,*abi*^*5*^/*UAS-HA-abi*,*Df* (*abi*^*5*^*/Df*, *HmlΔ > abi*), *UAS-HA-abi*^*Δ30–65*^/+; *HmlΔ*-*GAL4*,*abi*^*5*^/+,*Df* (*abi*^*5*^*/Df*, *HmlΔ > abi*^*Δ30–65*^), *HmlΔ*-*GAL4*,*abi*^*5*^/*UAS-HA-abi*^*W452K*^,*Df* (*abi*^*5*^*/Df*, *HmlΔ > abi*^*W452K*^), *HmlΔ-GAL4*,*abi*^*5*^/*UAS-HA-abi*^*4YF*^,*Df* (*abi*^*5*^*/Df*, *HmlΔ > abi*^*4YF*^), and *HmlΔ-GAL4*,*abi*^*5*^/*UAS-HA-abi*^*4YE*^,*Df* (*abi*^*5*^*/Df*, *HmlΔ > abi*^*4YE*^) late-third instar larvae, stained with anti-HA (pseudocolored magenta) and anti-P1(green) antibodies. **(B)** Quantification of the ratio of mean anti-HA to anti-P1 fluorescence intensities. Values are the percentages of the *abi*^*5*^*/Df*, *HmlΔ > abi* control. *n* = 9 cells. Data represent the mean ± SEM. Statistical analyses were performed using a one-way ANOVA with the Tukey–Kramer post hoc test (ns, not significant). Scale bar: 10 μm.

We then investigated the domain requirement for Abi function in mBSA uptake and crystal cell formation, a cellular readout of CME-mediated Notch activation. To this end, we performed genetic rescue experiments using UAS transgenes encoding WT HA-Abi, SCAR binding–defective HA-AbiΔ30–65, and WASp binding–defective HA-Abi-W452K (Kim et al., 2019). The *HmlΔ-*GAL4–driven expression of HA-Abi or HA-AbiΔ30–65 fully rescued the defect in mBSA uptake by primary *abi*^*5*^/*Df* hemocytes. In contrast, HA-Abi-W452K did not show any rescue activity (Fig. 5, B and C). Likewise, the *HmlΔ-GAL4*–driven expression of HA-Abi or HA-AbiΔ30–65, but not HA-Abi-W452K, restored the number of sessile crystal cells in *abi*^*5*^/*Df* larvae to WT levels (Fig. 5, D and E). The cortical submembrane localization and expression levels of the mutant transgenes were comparable to those of WT HA-Abi (Fig. S4). Together, these results suggest that the WASp-binding domain of Abi, but not its SCAR-binding domain, is specifically required during CME and crystal cell formation. Consistently, knockdown of WASp, but not SCAR, impaired mBSA uptake and crystal cell formation (Fig. 5, B–E).

Finally, we examined whether *abi* genetically interacts with *WASp* during CME and larval hematopoiesis. Uptake of mBSA by primary hemocytes from *abi*^*5*^/+, *WASp*^*3*^/+, and *SCAR*^*Δ37*^/+ larvae was normal compared with WT larvae (Fig. 5, F and G). Likewise, all these heterozygotes had normal numbers of sessile crystal cells (Fig. 5, H and I). Importantly, both mBSA uptake and crystal cell formation were significantly impaired by transheterozygous mutations of *abi* and *WASp*, but not by transheterozygous mutations of *abi* and *SCAR* (Fig. 5, F–I). Collectively, our results support a model in which Abi acts together with WASp to promote CME, which is required for Notch signaling activation and crystal cell formation.

### Abi physically interacts with Notch in a manner dependent on Ser-induced receptor ubiquitination

**Figure 6.**
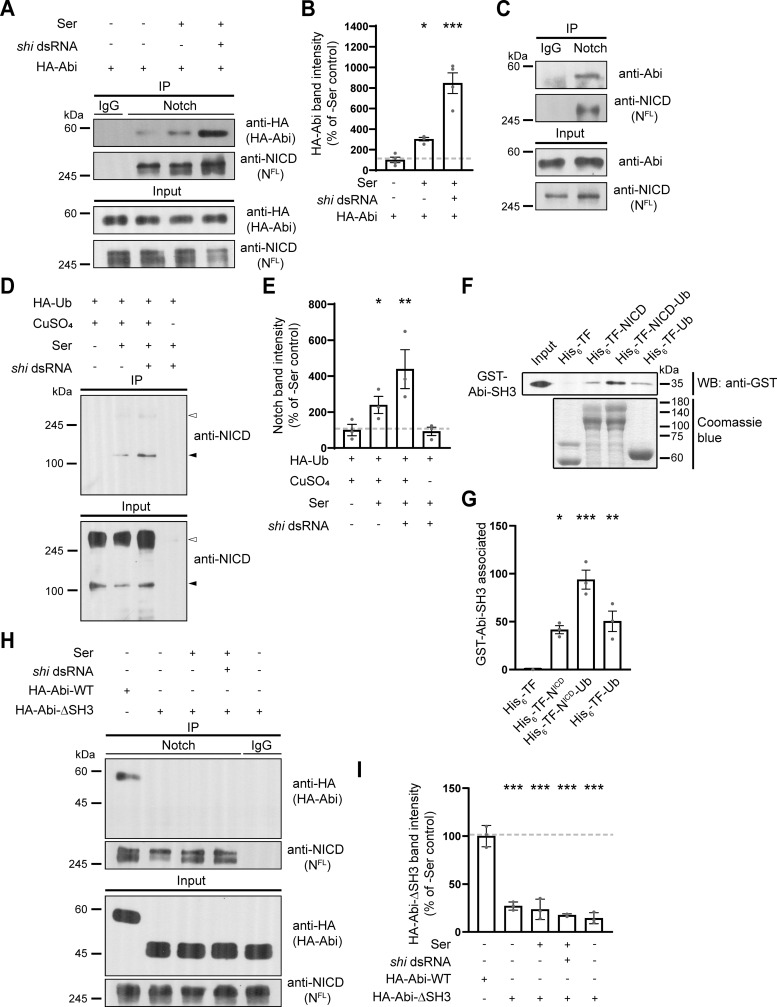
**Abi physically interacts with Notch in an Ub-dependent manner. (A and B)** Analysis of the Abi-Notch interaction by coimmunoprecipitation. S2N cells were transfected with HA-Abi cDNA with or without *shi* dsRNA, pretreated with 0.7 mM CuSO_4_ for 24 h, and further incubated in the presence and absence of S2S cells for 18 h. **(A)** Western blots of cell lysates (input) and anti-IgG or anti-NICD immunoprecipitates, probed with an anti-HA or anti-NICD antibody. **(B)** Quantification of HA-Abi levels in anti-NICD immunoprecipitates by densitometry. **(C)** Western blots of third instar larval lysates (input) and anti-IgG or anti-NICD immunoprecipitates, probed with an anti-Abi or anti-NICD antibody. **(D)** Ser-induced ubiquitination of Notch. Western blots of cell lysates (input) and anti-HA immunoprecipitates from S2N cells transfected with HA-Ub cDNA, with or without *shi* dsRNA treatment, probed with an anti-NICD antibody. Transfected cells were incubated in the presence or absence of S2S cells and CuSO_4_ for 18 h prior to western blotting. White and black arrowheads mark HA-Ub–modified full-length Notch and its breakdown product, respectively. **(E)** Quantification of cleaved Notch levels in anti-HA immunoprecipitates by densitometry. **(F and G)** Direct interaction between Abi and Notch in a Ub-dependent manner. Purified recombinant GST-Abi-SH3 was incubated with His_6_-TF, His_6_-TF-NICD, His_6_-TF-NICD-Ub, or His_6_-TF-Ub. **(F)** Western blot of His_6_ pull-downs probed with an anti-GST antibody (upper panel). The lower panel shows Coomassie blue staining of His_6_-TF proteins. **(G)** Quantification of GST-Abi-SH3 levels in His_6_ pull-downs by densitometry. **(H and I)** Analysis of the AbiΔSH3-Notch interaction. Cell lysates were prepared from S2N cells transfected with HA-Abi or HA-AbiΔSH3 cDNA, with or without *shi* dsRNA as in A. **(H)** Western blots of cell lysates (input) and anti-IgG or anti-NICD immunoprecipitates, probed with an anti-HA or anti-NICD antibody. **(I)** Quantification of HA-Abi and HA-AbiΔSH3 levels in anti-NICD immunoprecipitates by densitometry. Data represent the mean ± SEM. *n* = 3 independent experiments. Statistical analyses were performed using a one-way ANOVA with the Tukey–Kramer post hoc test (*P < 0.05; **P < 0.01; ***P < 0.001). Source data are available for this figure: SourceData F6.

To explore the Notch-specific role of Abi during CME, we examined the physical interaction between Abi and Notch. Notch coimmunoprecipitated with HA-Abi from S2N cell lysates (Fig. 6, A and B) and from third instar larval lysates (Fig. 6 C). The HA-Abi-Notch interaction in S2N cells was strongly enhanced by coculturing S2N cells with S2S cells (Fig. 6, A and B), showing that it was induced by Ser. Interestingly, blockade of endocytosis by knocking down Shi in S2N cells further increased the HA-Abi-Notch interaction (Fig. 6, A and B), indicating that Ser induces the Abi-Notch interaction at an early endocytic step.

Ligand-activated Notch is ubiquitinated before or during early endocytosis (Chastagner et al., 2017; Gupta-Rossi et al., 2004). Furthermore, a subset of SH3 domains in endocytic proteins can bind to ubiquitin (Ub) (Stamenova et al., 2007). These findings raise the possibility that Ser enhances the direct Abi-Notch interaction through Notch ubiquitination. To investigate this, we first attempted to confirm that Ser induces Notch ubiquitination in hemocyte-derived S2 cells. To this end, we overexpressed HA-Ub in S2N cells and analyzed anti-HA immunoprecipitates. Immunoblots probed with an anti-NICD antibody displayed a prominent band at ∼120 kDa, representing an HA-Ub–modified product of Notch cleavage (Lake et al., 2009). This HA-Ub–modified cleaved form of Notch was significantly upregulated by coculturing S2N cells with S2S cells and was further upregulated by knocking down Shi in S2N cells (Fig. 6, D and E), consistent with the conclusion that Ser-induced Notch ubiquitination occurs before or during early endocytosis. We then tested whether the SH3 domain of Abi mediates its direct interaction with Notch in a Ub-dependent manner. We performed pull-down experiments using purified GST-Abi-SH3 and His_6_-trigger factor–tagged NICD (His_6_-TF-NICD). GST-Abi-SH3 pulled down His_6_-TF-NICD but not control His_6_-TF (Fig. 6, F and G), showing that Abi-SH3 and NICD directly interact. Importantly, this interaction was enhanced by adding a single Ub molecule to His_6_-TF-NICD. GST-Abi-SH3 also interacted with His_6_-TF-Ub (Fig. 6, F and G), revealing the intrinsic ability of Abi-SH3 to bind to Ub. Finally, Notch failed to coimmunoprecipitate with HA-AbiΔSH3 from S2N cell lysates (Fig. 6, H and I), confirming the critical role of the Abi SH3 domain in mediating the Abi-Notch interaction. Collectively, these results suggest that Ser enhances the direct Abi-Notch interaction through receptor ubiquitination, thereby promoting Notch internalization via Abi-dependent CME.

### Opposing regulation of Notch-CME by Abl/PTP61F-mediated phosphorylation/dephosphorylation of Abi

Previous work in S2 cells demonstrated that Abl phosphorylates tyrosine residues at positions 148, 155, 248, and 285 of Abi and that these modifications can be reversed by the protein phosphatase PTP61F (Huang et al., 2007). Given the role of Abl-mediated Abi phosphorylation in macropinocytosis (Kim et al., 2019), we tested the relevance of Abl/PTP61F-mediated Abi phosphorylation/dephosphorylation in the regulation of CME and hemocyte homeostasis.

**Figure 7.**
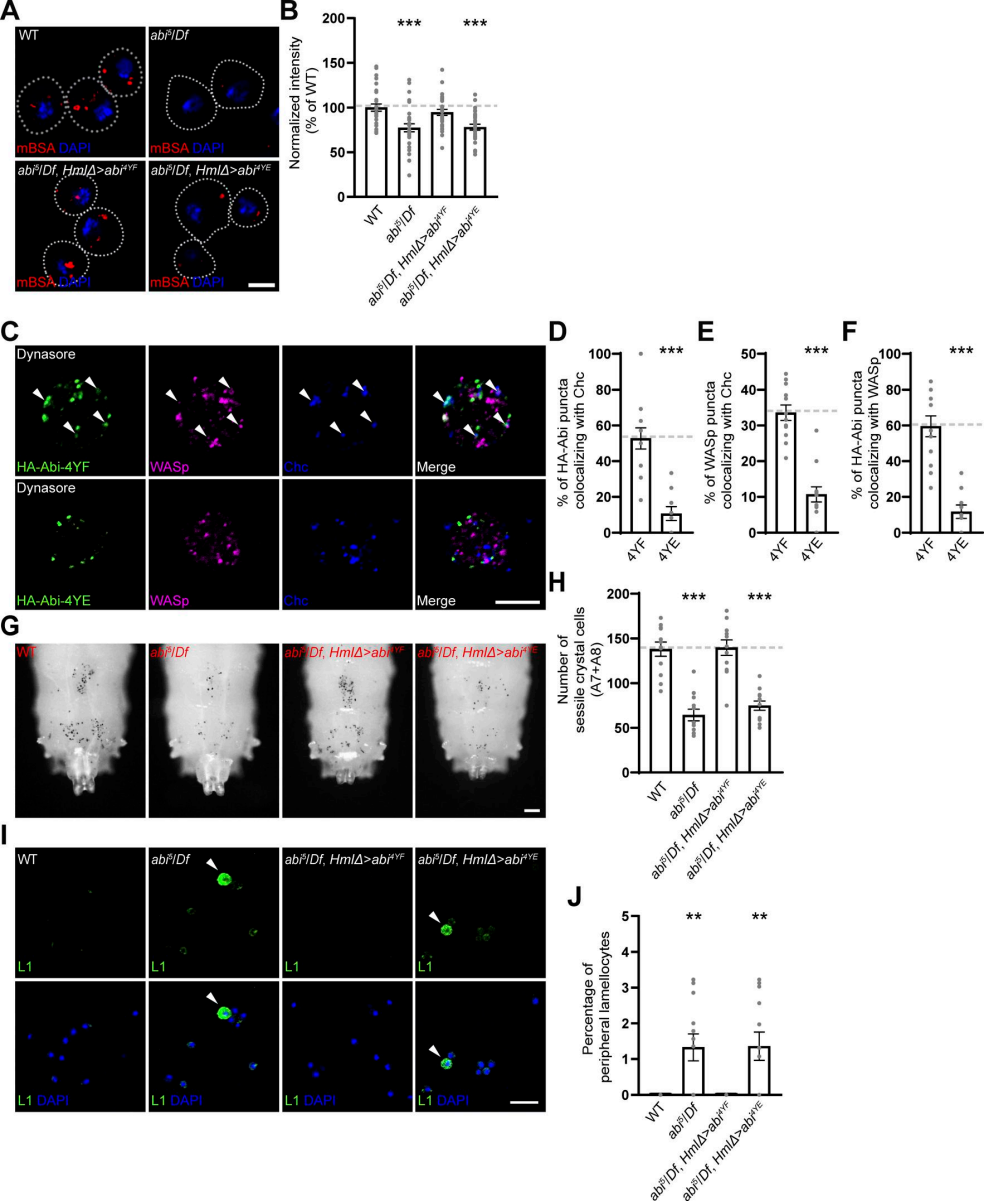
**Tyrosine phosphorylation of Abi inhibits its functions in CME and crystal cell development. (A)** Confocal images of primary hemocytes from WT, *abi*^*5*^/*Df*, *HmlΔ-GAL4*,*abi*^*5*^/*UAS-HA-abi*^*4YF*^,*Df* (*abi*^*5*^*/Df*, *HmlΔ > abi*^*4YF*^), and *HmlΔ-GAL4*,*abi*^*5*^/*UAS-HA-abi*^*4YE*^,*Df* (*abi*^*5*^*/Df*, *HmlΔ > abi*^*4YE*^) late-third instar larvae. Hemocytes were pulsed with Alexa Fluor 555-mBSA for 5 min, chased for 5 min, and stained with DAPI. **(B)** Quantification of the ratio of mean Alexa Fluor 555-mBSA to DAPI fluorescence intensities. Values are presented as percentages of *HmlΔ-GAL4*/+. *n* = 30 hemocytes. **(C)** Single confocal slices of primary hemocytes from *abi*^*5*^/*Df* larvae carrying *HmlΔ-GAL4* and *UAS-HA-Abi-4YF* or *UAS-HA-Abi-4YE*, pretreated with 100 μM dynasore for 30 min prior to immunofluorescence analysis using anti-HA (green), anti-WASp (pseudocolored magenta), and anti-Chc (blue) antibodies. **(D)** Quantification of HA-Abi-Chc colocalization. **(E)** Quantification of WASp-Chc colocalization. **(F)** Quantification of HA-Abi-WASp colocalization. **(G)** Bright-field images of heated (70°C, 10 min) late-third instar larvae of the indicated genotypes. **(H)** Number of heat-blackened crystal cells in the A7 and A8 segments. *n* = 12 larvae. **(I)** Confocal images of peripheral hemocytes from late-third instar larvae of indicated genotypes, stained with an anti-L1 antibody (green) and DAPI (blue). Arrowheads indicate L1^+^ lamellocytes. **(J)** Percentage of L1^+^ lamellocytes among all peripheral hemocytes (total DAPI count). Data represent the mean ± SEM. Statistical analyses are performed using a one-way ANOVA with the Tukey–Kramer post hoc test (**P < 0.01; ***P < 0.001). Scale bars: 5 μm (A); 10 μm (C); 20 μm (I); 200 μm (G).

We first compared the abilities of phospho-defective HA-Abi-4YF (Y148F + Y155F + Y248F + Y285F) and phospho-mimetic HA-Abi-4YE (Y148E + Y155E + Y248E + Y285E) to rescue the CME defect in *abi*^*5*^/*Df* hemocytes. When expressed under the control of *HmlΔ-GAL4*, HA-Abi-4YF fully restored the ability of *abi*^*5*^/*Df* hemocytes to efficiently uptake mBSA (Fig. 7, A and B). In contrast, HA-Abi-4YE did not display any rescue activity. Both HA-Abi-4YF and HA-Abi-4YE exhibited cortical submembrane localization and expression levels in primary hemocytes comparable to those of WT HA-Abi (Fig. S4), suggesting that Abl-mediated Abi phosphorylation negatively impacts CME. This conclusion was confirmed by comparing the colocalization of the two Abi variants with WASp and Chc at the submembrane cortex. HA-Abi-4YF was efficiently targeted together with WASp to Chc-labeled CCPs in *abi*^*5*^/*Df* primary hemocytes treated with dynasore (Fig. 7, C–F). In contrast, HA-Abi-4YE failed to colocalize with WASp or Chc (Fig. 7, C–F), implying that Abl-mediated phosphorylation of Abi impairs its ability to recruit WASp to CCPs and thereby perturbs CME.

We then compared the abilities of HA-Abi-4YF and HA-Abi-4YE to rescue the reduced crystal cell phenotype and the increased lamellocyte phenotype in *abi*^*5*^/*Df* larvae. HA-Abi-4YF restored the numbers of sessile crystal cells and circulating lamellocytes in *abi*^*5*^/*Df* mutants to WT levels, while HA-Abi-4YE did not display any rescue activity (Fig. 7, G–J). These results support the conclusion that Abl-mediated Abi phosphorylation impairs Notch-CME, thereby disrupting the formation of crystal cells and promoting lamellocyte differentiation.

**Figure 8.**
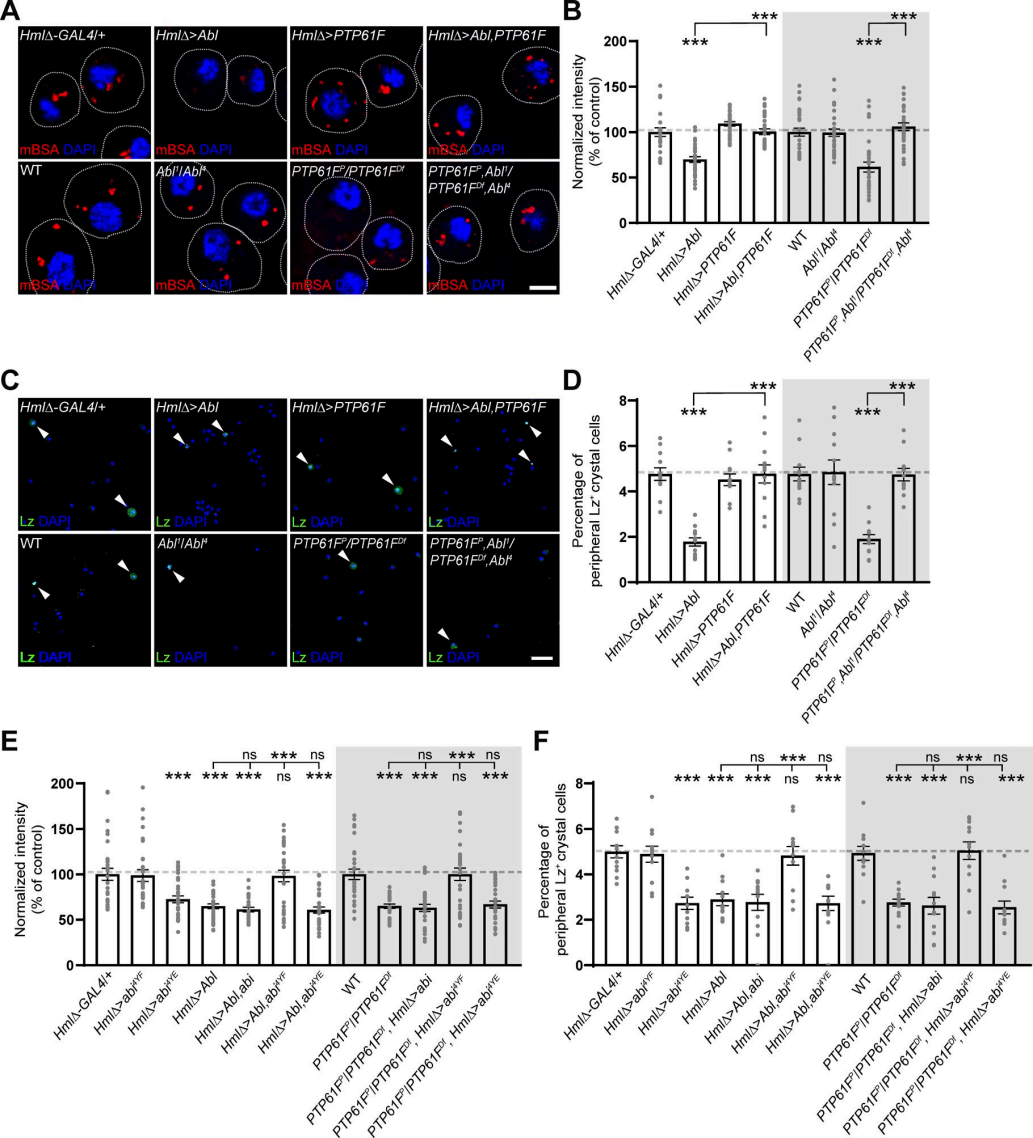
**Abl and PTP61F oppositely regulate CME and crystal cell formation by modulating the phosphorylation status of Abi. (A)** Single confocal sections of *HmlΔ-GAL4*/+, *UAS-Abl*/+; *HmlΔ-GAL4*/+ (*HmlΔ > Abl*), *HmlΔ-GAL4*/*UAS-PTP61F* (*HmlΔ > PTP61F*), *UAS-Abl*/+; *HmlΔ-GAL4*/*UAS-PTP61F* (*HmlΔ > Abl*, *PTP61F*), WT, *Abl*^*1*^/*Abl*^*4*^, *PTP61F*^*C05292*^/*Df(3L)BSC289* (*PTP61F*^*P*^/*PTP61F*^*Df*^), and *PTP61F*^*C05292*^,*Abl*^*1*^/*Df(3L)BSC289*,*Abl*^*4*^ (*PTP61F*^*P*^,*Abl*^*1*^/*PTP61F*^*Df*^,*Abl*^*4*^) late-third instar larvae. Hemocytes were pulsed with Alexa Fluor 555-mBSA for 5 min, chased for 5 min, and stained with DAPI. **(B)** Quantification of mBSA intensity normalized to DAPI intensity in primary hemocytes of the indicated genotypes. Values are presented as percentages of *HmlΔ-GAL4*/+ or WT. *n* = 30 hemocytes. **(C)** Confocal images of peripheral hemocytes from late-third instar larvae of the indicated genotypes, stained with anti-Lz (green) and DAPI (blue). **(D)** Percentage of Lz^+^ crystal cells among all peripheral hemocytes (total DAPI count) in the indicated genotypes. *n* = 12 larvae. **(E)** Quantification of mBSA intensity normalized to DAPI intensity in primary hemocytes of the following genotypes: *HmlΔ-GAL4*/+, *HmlΔ-GAL4*/*UAS-abi*^*4YF*^ (*HmlΔ > abi*^*4YF*^), *HmlΔ-GAL4*/*UAS-abi*^*4YE*^ (*HmlΔ > abi*^*4YE*^), *UAS-Abl*/+; *HmlΔ-GAL4*/+ (*HmlΔ > Abl*), *UAS-Abl*/+; *HmlΔ-GAL4*/*UAS-abi* (*HmlΔ > Abl*,*abi*), *UAS-Abl*/+; *HmlΔ-GAL4*/*UAS-abi*^*4YF*^ (*HmlΔ > Abl*,*abi*^*YF*^), *UAS-Abl*/+; *HmlΔ-GAL4*/*UAS-abi*^*4YE*^ (*HmlΔ > Abl*,*abi*^*YE*^), WT, *PTP61F*^*C05292*^/*Df(3L)BSC289* (*PTP61F*^*P*^/*PTP61F*^*Df*^), *PTP61F*^*C05292*^,*HmlΔ-GAL4*/*Df(3L)BSC289*,*UAS-abi* (*PTP61F*^*P*^/*PTP61F*^*Df*^, *HmlΔ > abi*), *PTP61F*^*C05292*^,*HmlΔ-GAL4*/*Df(3L)BSC289*,*UAS-abi*^*YF*^ (*PTP61F*^*P*^/*PTP61F*^*Df*^, *HmlΔ > abi*^*YF*^), and *PTP61F*^*C05292*^,*HmlΔ-GAL4*/*Df(3L)BSC289*,*UAS-abi*^*YE*^ (*PTP61F*^*P*^/*PTP61F*^*Df*^, *HmlΔ > abi*^*YE*^). *n* = 30 hemocytes. **(F)** Quantification of Lz^+^ crystal cells among all peripheral hemocytes (total DAPI count) in the indicated genotypes. *n* = 12 larvae. Data represent the mean ± SEM. Comparisons are with *HmlΔ-GAL4*/+ or WT unless otherwise indicated (***P < 0.001; ns, not significant). Statistical analyses were performed using a one-way ANOVA with the Tukey–Kramer post hoc test. Scale bars: 5 μm (A); 20 μm (C).

We also examined the functional interaction between Abl and PTP61F during CME in primary hemocytes. The overexpression of Abl strongly impaired the ability of hemocytes to uptake mBSA (Fig. 8, A and B). Importantly, the overexpression of PTP61F had no effect on mBSA uptake by hemocytes but completely suppressed the reduction of mBSA uptake due to the overexpression of Abl (Fig. 8, A and B). The antagonistic relationship between Abl and PTP61F was confirmed in a separate set of experiments using loss-of-function mutations in *Abl* and *PTP61F*. Similar to the overexpression of Abl, loss of PTP61F impaired the ability of hemocytes to uptake mBSA (Fig. 8, A and B). This phenotype was completely suppressed by loss of Abl, which by itself had no effect on mBSA uptake by hemocytes (Fig. 8, A and B). Thus, a reciprocally antagonistic relationship exists between Abl and PTP61F in the regulation of CME.

**Figure S5.**
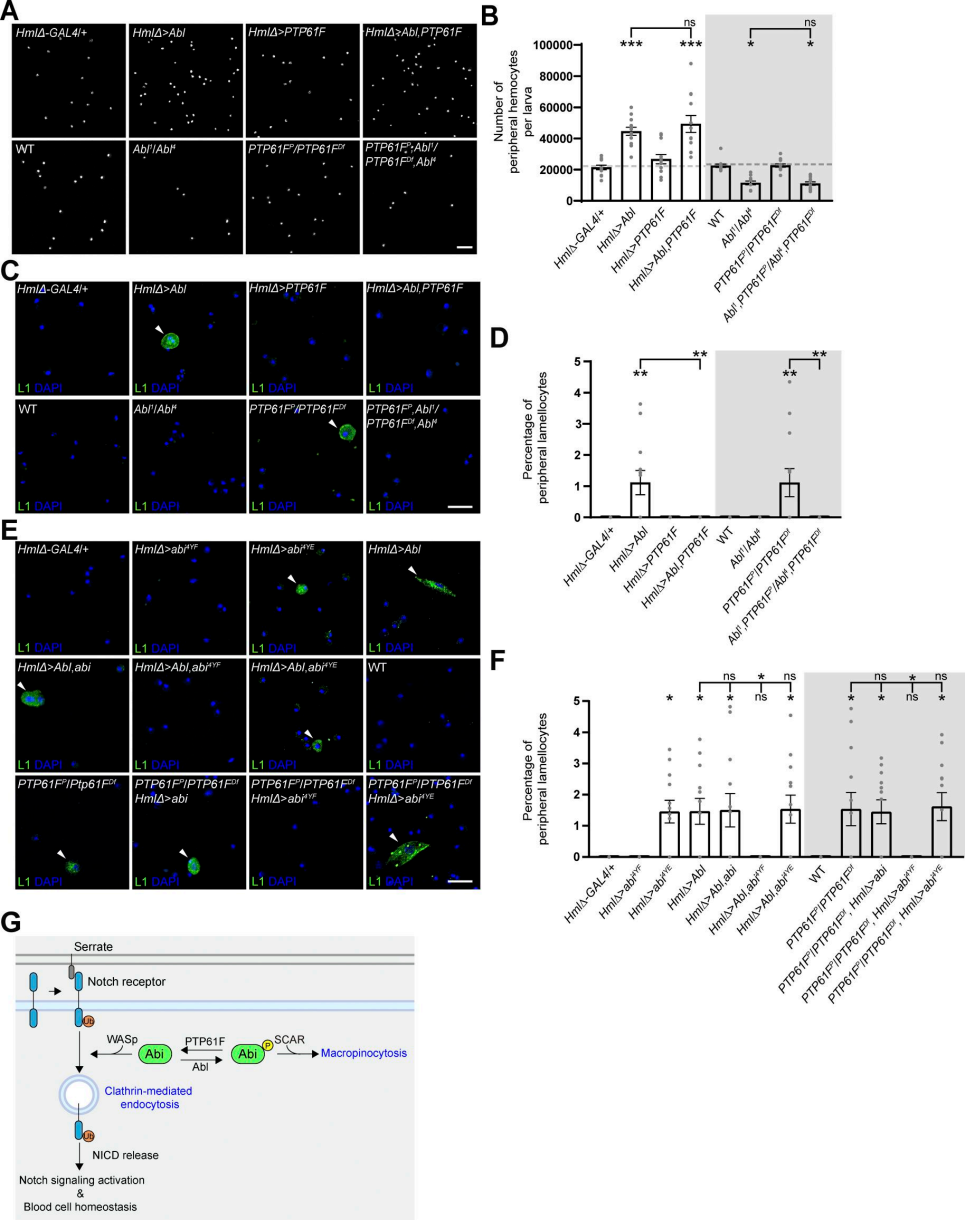
**Abl regulates hemocyte homeostasis independently of PTP61F. (A)** Confocal images of DAPI-stained peripheral (circulating and sessile) hemocytes from late-third instar larvae of the following genotypes: *HmlΔ-GAL4*/+, *UAS-Abl*/+; *HmlΔ-GAL4*/+ (*HmlΔ > Abl*), *HmlΔ-GAL4*/*UAS-PTP61F* (*HmlΔ > PTP61F*), *UAS-Abl*/+; *HmlΔ-GAL4*/*UAS-PTP61F* (*HmlΔ > Abl, PTP61F*), WT, *Abl*^*1*^/*Abl*^*4*^, *PTP61F*^*C05292*^/*Df(3L)BSC289* (*PTP61F*^*P*^/*PTP61F*^*Df*^), and *PTP61F*^*C05292*^,*Abl*^*1*^/*Df(3L)BSC289*,*Abl*^*4*^ (*PTP61F*^*P*^, *Abl*^*1*^/*PTP61F*^*Df*^,*Abl*^*4*^). **(B)** Number of peripheral hemocytes per larva. *n* = 12 larvae. **(C)** Confocal images of peripheral hemocytes from late-third instar larvae in the indicated genotypes shown in B, stained with anti-L1 (green) and DAPI (blue). Arrowheads indicate L1^+^ lamellocytes. **(D)** Percentage of L1^+^ lamellocytes among all peripheral hemocytes (total DAPI count). **(E)** Confocal images of peripheral hemocytes from late-third instar larvae in the indicated genotypes shown in Fig. 8 F, stained with anti-L1 (green) and DAPI (blue). Arrowheads indicate L1^+^ lamellocytes. **(F)** Percentage of L1^+^ lamellocytes among all peripheral hemocytes. **(G)** Model for the opposite regulation of CME-mediated Notch signaling activation and blood cell homeostasis by Abl-mediated Abi phosphorylation and PTP61F-mediated dephosphorylation. Data represent the mean ± SEM. Comparisons are with *HmlΔ-GAL4*/+ unless otherwise indicated. Statistical analyses were performed using a one-way ANOVA with the Tukey–Kramer post hoc test (*P < 0.05; **P < 0.01; ***P < 0.001; ns, not significant). Scale bars: 20 μm.

Next, we tested the antagonistic *Abl*-*PTP61F* interaction in the regulation of hemocyte homeostasis, focusing on larval peripheral hemocytes. The *HmlΔ-GAL4–*driven overexpression of Abl in WT larvae dramatically increased the total number of hemocytes but significantly reduced the frequency of Lz^+^ crystal cells (Fig. 8, C and D; and Fig. S5, A and B). Concurrently, Abl overexpression also increased the frequency of L1^+^ lamellocytes (Fig. S5, C and D). In contrast, the *HmlΔ-GAL4*–driven overexpression of PTP61F had no effect on the total hemocyte, crystal cell, and lamellocyte populations (Fig. 8, C and D; and Fig. S5, A–D). Notably, the co-overexpression of Abl and PTP61F restored the frequencies of crystal cells and lamellocytes to WT levels, while the total hemocyte number remained elevated (Fig. 8, C and D; and Fig. S5, A–D), indicating that Abl and PTP61F act antagonistically specifically during crystal cell and lamellocyte formation. We extended this analysis by using loss-of-function mutations in *Abl* and *PTP61F*. Loss of PTP61F did not alter the total number of peripheral hemocytes (Fig. S5, A and B). However, it decreased the frequency of Lz^+^ crystal cells but increased the frequency of lamellocytes (Fig. 8, C and D; and Fig. S5, C and D). In contrast, loss of Abl significantly decreased the total number of peripheral hemocytes without affecting crystal cell and lamellocyte frequencies (Fig. 8, C and D; and Fig. S5, A–D). Importantly, the reduced crystal cell and increased lamellocyte phenotypes in *PTP61F* mutants were completely suppressed by loss of *Abl* (Fig. 8, C and D; and Fig. S5, C and D), confirming the antagonistic regulatory relationship between Abl and PTP61F during crystal cell and lamellocyte formation. However, loss of *PTP61F* did not alter the underproduction of hemocytes in *Abl* mutants (Fig. S5, A and B), implying that Abl can also regulate plasmatocyte development independently of PTP61F.

Finally, we asked whether the defective CME and hematopoietic abnormalities observed in Abl-overexpressing larvae and *PTP61F* mutants result from increased phosphorylation of Abi at the tyrosine residues targeted by Abl and PTP61F. The *HmlΔ-GAL4*–driven overexpression of HA-Abi-4YF in Abl-overexpressing larvae and *PTP61F* mutants fully rescued the defects in CME, crystal cell formation, and lamellocyte development, while HA-Abi and HA-Abi-4YE had no effect (Fig. 8, E and F; and Fig. S5, E and F). These results are consistent with a model in which Abl and PTP61F reciprocally regulate CME and hemocyte homeostasis by modulating the phosphorylation status of Abi (Fig. S5 G).

## Discussion

Abi proteins, which were originally identified as substrate adaptor proteins for the Abl kinase (Dai and Pendergast, 1995; Juang and Hoffmann, 1999; Shi et al., 1995), play a key role in the regulation of SCAR and WASp activities in distinct actin-based processes (Bogdan et al., 2005; Innocenti et al., 2005). Although disruption of mammalian Abi1 has been implicated in the pathogenesis of myeloproliferative neoplasm and acute myeloid leukemia (Chorzalska et al., 2018; Morerio et al., 2002; Shibuya et al., 2001; Taki et al., 1998), it is unknown how actin-regulatory Abi proteins regulate normal hematopoiesis. Here, we present data indicating that *Drosophila* Abi and WASp, but not SCAR, play an important role in maintaining blood cell homeostasis by promoting CME-mediated Notch signaling activation. Furthermore, our findings indicate that the role of Abi in Notch-CME is negatively and positively regulated by Abl-mediated Abi phosphorylation and PTP61F-mediated Abi dephosphorylation, respectively. The study identifies Abi as a key regulator that links actin cytoskeletal dynamics to Notch signaling and blood cell homeostasis, controlled by a reversible phosphorylation switch involving Abl kinase and PTP61F phosphatase (Fig. S5 G).

*Drosophila* larval plasmatocytes are highly plastic cells that can transdifferentiate into crystal cells and lamellocytes (Csordás et al., 2021). Interestingly, these two distinct routes of plasmatocyte transdifferentiation are oppositely regulated. In healthy larvae, plasmatocytes transdifferentiate only into crystal cells (Honti et al., 2010; Leitao and Sucena, 2015; Marcetteau et al., 2025). In contrast, plasmatocytes are activated to transdifferentiate into lamellocytes upon wasp infection (Anderl et al., 2016; Honti et al., 2010; Stofanko et al., 2010), a condition in which the crystal cell fate is repressed (Krzemien et al., 2010). This study provides data indicating that Abi acts in plasmatocytes to favor adoption of a crystal cell fate over a lamellocyte fate in the larval peripheral hematopoietic compartment. We show that *abi*^*5*^/*Df* larvae contain fewer crystal cells but more lamellocytes than WT larvae. These phenotypes are completely rescued by the re-expression of Abi using plasmatocyte-specific *HmlΔ-GAL4* and *eater-GAL4*, but not crystal cell–specific *lz-GAL4* or lamellocyte-specific *MSNF9-GAL4*, supporting the notion that Abi acts in plasmatocytes to promote their transdifferentiation into crystal cells. Lineage tracing analyses using *HmlΔ-GAL4* confirm a cell-autonomous requirement for Abi in Hml^+^ plasmatocytes in the generation of larval peripheral crystal cells.

Previous work suggests that transdifferentiation of Hml^+^ plasmatocytes is sufficient to explain the expansion of peripheral crystal cells during larval development (Leitao and Sucena, 2015). In contrast, we found that a sizable portion of these cells are not marked by the G-TRACE system driven by *HmlΔ-GAL4*, suggesting the presence of an *Hml* lineage–independent pathway contributing to crystal cell formation. Notably, a previous study identified a small population (<1% of circulating hemocytes) of Wg^+^Hml^−^ hemocyte precursors in circulation (Sinenko et al., 2010), raising the possibility that a progenitor-based mechanism contributes, at least in part, to the expansion of peripheral crystal cells. Future studies will be needed to determine whether the *Hml* lineage–independent and Abi-insensitive pathway reflects straightforward differentiation of residual precursor cells or represents an alternative mode of plasmatocyte transdifferentiation distinct from the classical Hml^+^ plasmatocyte–dependent mechanism.

Our lineage tracing experiments further indicate that in addition to promoting transdifferentiation of *Hml* lineage–traced plasmatocytes, Abi is required for the maintenance of *Hml* non-lineage–traced crystal cells in the LG. Consequently, the reduced crystal cell phenotype observed in *abi* mutant LGs reflects loss of both functions. Strikingly, this phenotype is fully rescued by the re-expression of HA-Abi using *HmlΔ-GAL4*, although *Hml* non-lineage–traced crystal cells constitute the predominant subpopulation in the LG. This finding suggests that Abi may act non–cell-autonomously in Hml^+^ plasmatocytes to support the survival of neighboring crystal cells. Alternatively, or in addition, *Hml* lineage–traced plasmatocytes may acquire increased plasticity, thereby compensating for the loss of *Hml* non-lineage–traced crystal cells whose survival depends on the cell-autonomous function of Abi.

Ser-dependent Notch signaling promotes transdifferentiation of Hml^+^Lz^−^ plasmatocytes into Hml^−^Lz^+^ crystal cells via the intermediate Hml^+^Lz^+^ state while repressing the lamellocyte fate (Leitao and Sucena, 2015; Small et al., 2014). Here, we provide multiple lines of evidence that Abi maintains hemocyte homeostasis by activating Ser-dependent Notch signaling. First, loss of Abi reduces Notch reporter (NRE-GFP) expression in Hml^+^Lz^−^ and Hml^+^Lz^+^ cells. Second, *abi* displays dosage-sensitive genetic interactions with *N* and *Ser*, but not *Dl*, during crystal cell and lamellocyte development. Third, the overexpression of constitutively active Notch (i.e., NICD) suppresses hematopoietic defects in an *abi* mutant background. These observations provide strong evidence that Abi regulates crystal cell/lamellocyte differentiation by activating Notch signaling in Hml^+^Lz^−^ plasmatocytes.

How does Abi activate Notch signaling? Notch is internalized via multiple endocytic routes (Windler and Bilder, 2010). In *Drosophila* and mammals, ligand-dependent activation of Notch receptors requires receptor internalization via CME (Chapman et al., 2016; Windler and Bilder, 2010). We provide evidence that Abi activates Ser-dependent Notch signaling by promoting Notch-CME via two distinct mechanisms: local actin polymerization and Notch recruitment to sites of CME. In support of the former mechanism, Abi and WASp, but not SCAR, are recruited to Chc-labeled CCPs that are stabilized by pharmacological inhibition of dynamin. In addition, the WASp-binding domain, but not the SCAR-binding domain, of Abi is essential for CME. Furthermore, *abi* and *WASp*, but not *SCAR*, display a transheterozygous interaction that produces defects in CME and crystal cell formation, the latter serving as a readout of CME-mediated Notch activation. These results suggest that Abi promotes CME by targeting WASp-dependent actin polymerization at CCPs and are consistent with the previously reported finding that mammalian N-WASp is required for CME (Hinze and Boucrot, 2018). This work also provides evidence that Abi plays a Notch-specific role during CME. We found that the Abi SH3 domain directly interacts with NICD in a Ub-dependent manner. Additionally, we found that Ser induces NICD ubiquitination at an early step of Notch endocytosis, extending the previous finding that ligand-activated mammalian Notch1 undergoes ubiquitination mediated by the E3 Ub ligase Deltex 4 before it is endocytosed (Chastagner et al., 2017). Based on these findings, we propose that Abi facilitates Notch-CME by providing important links between the CME machinery, WASp, and ubiquitinated Notch.

Abi acts together with SCAR, but not WASp, to promote Rac1-dependent lamella formation and macropinocytosis (Innocenti et al., 2005; Kim et al., 2019). Lamella formation and macropinocytosis are positively and negatively regulated by Abl-mediated Abi phosphorylation and PTP61F-mediated Abi dephosphorylation, respectively (Huang et al., 2007; Kim et al., 2019). Given our conclusion that Abi and WASp, but not SCAR, are essential for CME, an interesting question is whether reciprocal regulation of Abi phosphorylation by Abl and PTP61F also plays a key role in setting the balance between WASp and SCAR during CME. Several of our findings indicate this is the case. First, a phospho-defective form of Abi (Abi-4YF), but a phospho-mimetic form (Abi-4YE), localizes together with WASp to sites of CME and rescues both the CME defect and the decreased crystal cell and increased lamellocyte phenotypes of *abi*^*5*^/*Df* mutants. Second, the overexpression of Abl or loss of PTP61F causes CME and hematopoietic defects similar to those seen in *abi* mutants. Furthermore, these Abl gain-of-function and PTP61F loss-of-function phenotypes are rescued by the overexpression of PTP61F and loss of Abl, respectively, suggesting that the balance between Abl kinase and PTP61F phosphatase activity is important for proper CME and for determining crystal cell versus lamellocyte fate. Finally, the defects associated with the overexpression of Abl or loss of PTP61F are suppressed by the overexpression of Abi-4YF but not Abi-4YE, supporting a functional link between Abi and the Abl/PTP61F switch. Together with the demonstrated role of Abl-mediated Abi phosphorylation and SCAR in macropinocytosis (Innocenti et al., 2005; Kim et al., 2019), these findings demonstrate that reciprocal regulation of Abi phosphorylation by Abl and PTP61F is a key mechanism that differentially regulates the activities of the Abi-SCAR and Abi-WASp modalities during two modes of endocytosis: macropinocytosis and CME. It will be interesting to investigate how the phosphorylation status of Abi influences its interactions with WASp and SCAR, as well as its recruitment to sites of CME and macropinocytosis.

In addition to its role in Notch endocytosis, Abl also regulates endosomal trafficking of internalized receptors to attenuate Notch signaling, thereby ensuring the maintenance of neuronal cell fate in the developing eye and the proper patterning of wing veins (Miranda-Alban et al., 2025; Xiong et al., 2013). In this context, Abl phosphorylates Notch at the PPxY motif within the NICD (Miranda-Alban et al., 2025), which serves as the binding site for Nedd4-family E3 Ub ligases (Jennings et al., 2007). This phosphorylation is proposed to enhance the binding of Nedd4-family ligases, thereby promoting polyubiquitination of internalized Notch and its subsequent incorporation into the intraluminal vesicles of late endosomes/multivesicular bodies, ultimately leading to receptor downregulation and signal attenuation (Schnute et al., 2018; Shimizu et al., 2014). Nevertheless, Abl-dependent regulation of Notch via Nedd4-family ligases, including Suppressor of Deltex and Nedd4Lo, has been demonstrated primarily in ligand-independent signaling contexts (Miranda-Alban et al., 2025; Shimizu et al., 2014; Xiong et al., 2013). Thus, Abl likely employs distinct regulatory mechanisms to fine-tune ligand-dependent and ligand-independent Notch signaling.

In conclusion, this study uncovers a previously unrecognized role of Abi in coupling actin dynamics to ligand-dependent Notch activation via CME during blood cell differentiation. This process is promoted by PTP61F-mediated Abi dephosphorylation but antagonized by Abl-mediated Abi phosphorylation, revealing a reversible phosphorylation switch that links actin remodeling, endocytosis, and Notch signaling. These findings provide a conceptual framework for understanding the Abl-independent function of mammalian Abi in normal hematopoiesis, distinct from its role in BCR-Abl–driven leukemogenesis. Given the context-dependent tumor-suppressive and oncogenic functions of Notch signaling in hematopoiesis (Gu et al., 2016; Kushwah et al., 2014), our results also establish a foundation for exploring analogous Abl/PTP1B-Abi-Notch regulatory mechanisms in mammalian hematopoiesis and leukemia.

## Materials and methods

### *Drosophila* stocks

Flies were maintained at 25°C on standard cornmeal medium. *w*^*1118*^ was used as the WT control. The following fly stocks have been previously described (Kim et al., 2019): *abi*^*5*^, *UAS-HA-abi*, *UAS-HA-abi*^*Δ30–65*^, *UAS-HA-abi*^*W452K*^, *UAS-HA-abi*^*4YF*^, and *UAS-HA-abi*^*4YE*^. *UAS-abi*^*RNAi*^ was generated in the *w*^*1118*^ background using standard procedures. *HmlΔ*-*DsRed* was a gift from K. Brückner (University of California, San Francisco, CA, USA). The following fly stocks were obtained from the Bloomington Stock Center: *N*^*55e11*^ (RRID: BDSC_28813), *Ser*^*RX82*^ (RRID: BDSC_6300), *Dl*^*B2*^ (RRID: BDSC_5602), *Abl*^*1*^ (RRID: BDSC_3554), *Abl*^*4*^ (RRID: BDSC_3553), *PTP61F*^*C05292*^ (RRID: BDSC_17698), *Df(3R)su(Hw)7* (RRID: BDSC_1049; a deficiency covering the *abi* locus), *Df(3L)EXEL7321* (RRID: BDSC_7977), *Df(3L)BSC289* (RRID: BDSC_23674; a deficiency covering the *PTP61F* locus), *NRE-GFP* (Saj et al., 2010), *UAS-mCD8-GFP* (RRID: BDSC_30002), *UAS-Ser* (RRID: BDSC_5815), *UAS-N* (RRID: BDSC_26820), *UAS-NICD* (RRID: BDSC_94074), *UAS-shi*^*RNAi*^ (RRID: BDSC_28513), *UAS-abi*^*RNAi*^ (RRID: BDSC_51455), *UAS-Rabankyrin*^*RNAi*^ (RRID: BDSC_34883), *UAS-Chc*^*RNAi*^ (RRID: BDSC_34742), *UAS-WASp*^*RNAi*^ (RRID: BDSC_25955), *UAS-SCAR*^*RNAi*^ (RRID: BDSC_31126), *UAS-Abl* (RRID: BDSC_28993), *UAS-PTP61F* (RRID: BDSC_56194), and *UAS-G-TRACE* (RRID: BDSC_28280; *UAS-Flp*, *UAS-RedStinger*, *ubi-p63E[FRT.stop]Stinger*). The following GAL4 lines were used to drive the expression of UAS transgenes: *HmlΔ-GAL4* (Sinenko and Mathey-Prevot, 2004), *eater-GAL4* (Tokusumi et al., 2009), *lz-GAL4* (Lebestky et al., 2000), *MSNF9-GAL4* (Tokusumi et al., 2009), and *Pxn-GAL4* (Stramer et al., 2005), *srp-GAL4* (Bruckner et al., 2004).

To analyze the temporal window of Abi requirement, embryos were collected for 6 h and allowed to further develop for 57 h (60 h AEL), 75 h (78 h AEL), 89 h (92 h AEL), or 109 h (112 h AEL) at 25°C. The developmental stage of each larva was confirmed based on morphological characteristics, as previously described (Vaufrey et al., 2018).

### Molecular biology

To generate *UAS-abi*^*RNAi*^, an *abi* cDNA fragment spanning nucleotides 1–240 was PCR-amplified from the cDNA clone LD37010 (*Drosophila* Genomics Resources Center [DGRC]) and cloned as an inverted repeat into the pWIZ vector (Lee and Carthew, 2003).

For RNAi experiments in S2 cells or S2-Mt-N cells stably expressing Notch (S2N cells), *abi*, *shi*, *Chc*, and *Rabankyrin* double-stranded RNA (dsRNA) were synthesized by in vitro transcription of their cognate DNA templates using MEGAscript T7 Transcription Kit (Thermo Fisher Scientific). DNA templates were generated by PCR using primers containing the T7 promoter sequence upstream of the following sequences: *abi*, 5′-GCC​TCG​CAT​CGA​TAT​TCT​A-3′ and 5′-ACC​ATA​TAG​AGC​GTA​TGT​G-3′; *shi*, 5′-GGA​GTT​ACC​GAA​TAT​GGC-3′ and 5′-ATC​TAT​TCA​CCA​CGC​CAA-3′; *Chc*, 5′-GCC​TGC​TGG​AAA​TGA​AT-3′ and 5′-CGC​TCC​ACC​TCC​TTA​AT-3′; and *Rabankyrin*, 5′-GCC​AAA​TCT​AGT​TAA​GAA​G-3′ and 5′-GCA​GCG​GAG​ATG​CCT​TAT​C-3′.

For transient expression in S2N cells, the cDNA insert of *pAc-HA-abi* and *pAc-HA-abiΔSH3* was moved into the pMT vector (Invitrogen) to produce *pMT-HA-abi* and *pMT-HA-abiΔSH3*, respectively (Kim et al., 2019). *HA-Ub* cDNA encoding Ub with an HA epitope was generated by PCR-based mutagenesis and cloned into the pMT vector to produce *pMT-HA-Ub*. *NRE-GFP* cDNA was PCR-amplified from genomic DNA of *NRE-GFP* flies and inserted into the pTOP-V2 vector (Enzynomics) to produce *pTOP-NRE-GFP*. For stable expression in S2 cells, a full-length Ser cDNA clone (Clone ID: RE42104) was obtained from DGRC, and a Myc epitope was introduced in-frame immediately downstream of the signal-peptide sequence via PCR-based mutagenesis. The resulting *Myc-Ser* cDNA was ligated into the pTOP-V2 vector and then moved into the Ac5-STABLE1-neo vector (Addgene) to produce *Ac5-Myc-Ser-STABLE1-Neo*.

For expression in *Escherichia coli*, a cDNA encoding NICD (amino acid residues 1765–2703) was PCR-amplified from genomic DNA isolated from *UAS-N* transgenic flies and cloned into the 6xHis tag vector pCold-TF (pCold-His6-TF, Takara) to produce *pCold-His*_*6*_*-TF-NICD*. cDNA encoding Ub was PCR-amplified and cloned into the pCold-His_6_-TF and pCold-His_6_-TF-NICD vectors to produce *pCold-His*_*6*_*-TF-Ub* and *pCold-His*_*6*_*-TF-NICD-Ub*, respectively. *pGEX-Abi-SH3* was previously described (Kim et al., 2019).

### Cell culture and transfection

*Drosophila* S2 and S2N cells were obtained from DGRC. S2 cells stably expressing the Myc-Ser fusion under the control of an actin promoter (S2S cells) were generated by transfecting the *Ac5-Myc-Ser-STABLE1-Neo* vector and selecting cells in 2 mg/ml G418 (Invitrogen) as previously described (Kim et al., 2024). These cells were maintained at 25°C in Shields and Sang M3 Insect Medium (USBiological Life Sciences) supplemented with 10% heat-inactivated (30 min, 56°C) fetal bovine serum, 1 g/liter yeast extract, 2.5 g/liter Bacto-peptone, 100 U/ml penicillin, and 100 μg/ml streptomycin (Gibco). S2N and S2S cells were maintained in the presence of 0.2 μM methotrexate (Sigma-Aldrich) and 2 mg/ml G418 (InvivoGen), respectively.

S2 cells were transfected in serum-free medium using Cellfectin (Invitrogen), following the manufacturer’s instructions. Typically, 2 × 10^6^ cells were transfected with 2 μg of plasmid DNA or 5 μg of dsRNA.

### Binding experiments

To analyze complex formation between Notch and Abi during endocytosis, S2N cells were transfected with either *pMT*-*HA*-*Abi* or *pMT-HA-abiΔSH3*, in the presence or absence of *shi* dsRNA. At 24 h after transfection, cells were pretreated with 0.7 mM CuSO_4_ in M3 medium for 24 h to induce Notch expression. Pretreated S2N cells (2 × 10^7^ cells) were cocultured with S2S cells (1 × 10^7^ cells) for 18 h in a 6-well plate. Cocultured cells were harvested, washed in phosphate-buffered saline (PBS), and subjected to repeated freeze–thaw cycles in 500 μl of lysis buffer (50 mM Tris-HCl, pH 7.5, 0.2% Triton X-100, 125 mM NaCl, 5% glycerol, and protease inhibitors) for 30 min. Following centrifugation at 12,000 × *g* for 15 min at 4°C, supernatants were precleared by incubation with protein A/G PLUS Agarose (Santa Cruz Biotechnology) for 1 h at 4°C. The samples were then incubated with 1 μg of mouse anti-NICD antibody (DSHB) overnight and then with protein A/G PLUS Agarose for 2 h at 4°C. Beads were washed three times with lysis buffer and boiled in SDS sample buffer. Samples were analyzed by 8% SDS-PAGE and western blotting as previously described (Kim et al., 2017). The following antibodies were used: rabbit anti-HA (1:1,000, C29F4, Cell Signaling Technology) and mouse anti-NICD (1:500, C17.9C6, DSHB).

To analyze the direct interaction between Abi and Notch, GST-Abi-SH3, His_6_-TF, His_6_-TF-NICD, His_6_-TF-NICD-Ub, and His_6_-TF-Ub were expressed in *E. coli* and purified using glutathione-Sepharose 4B (GE Healthcare) or HisPur Ni-NTA (Thermo Fisher Scientific) beads. GST-Abi-SH3 (1 μg) was incubated with each His_6_-TF-tagged protein (1 μg) for 3 h in 500 μl of binding buffer (40 mM Tris-HCl, pH 7.5, 0.25 M KCl, 0.02% Triton X-100, 10% glycerol, and 0.5 mg/ml BSA). Mixtures were then incubated with HisPur Ni-NTA beads to capture protein complexes. Beads were washed five times with binding buffer and boiled in SDS sample buffer. The eluates were analyzed by 8% SDS-PAGE and western blotting using a mouse anti-GST antibody (1:1,000, 26H1; Cell Signaling Technology).

### Notch ubiquitination assay

S2N cells were transfected with *pMT*-*HA-Ub* in the presence or absence of *shi* dsRNA. At 24 h after transfection, cells were pretreated with 0.7 mM CuSO_4_, cocultured with S2S cells, and subjected to immunoprecipitation using an anti-HA antibody, as described above. The immunoprecipitates were analyzed by 8% SDS-PAGE and western blotting using a mouse anti-NICD antibody.

### Immunostaining

S2 cells and primary hemocytes were fixed in ice-cold PBS containing 4% formaldehyde for 10 min, as previously described (Anderl et al., 2016). After washing with PBS, cells were permeabilized in PBST-0.2 (PBS containing 0.2% Triton X-100) for 10 min and incubated in blocking buffer (PBS containing 0.2% BSA) for 10 min. Cells were then incubated with primary antibodies diluted in blocking buffer (2 h at 25°C or overnight at 4°C). After washing with PBS, cells were stained with fluorescent secondary antibodies diluted in blocking buffer for 1 h. The following primary antibodies were used: mouse anti-P1 (1:50, a gift from I. Ando), mouse anti-L1 (1:30, a gift from I. Ando), mouse anti-Lz (1:10, anti-Lozenge, DSHB), mouse anti-Antp (1:10, 8C11, DSHB), rabbit anti-Avl (1:1,000, Haberman et al., 2010); rat anti-Abi (1:100, Kim et al., 2019), rabbit anti-Chc (1:1,000, ab59710; Abcam), rabbit anti-Myc (1:200, 71D10; Cell Signaling Technology), chicken anti-GFP (1:500, 600-901-215; Rockland), mouse anti-WASp (1:100, P5E1-WASp; DSHB), mouse anti-SCAR (1:100, P1C1-SCAR; DSHB), and rat anti-HA (1:200, 3F10; Roche). The following secondary antibodies from Jackson ImmunoResearch Laboratories were used at a dilution of 1:200: FITC-, Cy3-, and Cy5-conjugated donkey anti-mouse (catalog numbers 715-095-150, 715-165-150, and 715-175-150, respectively); FITC- and Cy5-conjugated donkey anti-rabbit (711-095-152 and 711-175-152, respectively); FITC- and Cy5-conjugated donkey anti-rat (712-095-153 and 712-175-150, respectively); and FITC-conjugated goat anti-chicken (703-095-155). Stained cells were mounted using Vectashield antifade mounting medium (Vector Laboratories) and imaged using a Zeiss LSM 800 microscope equipped with a Plan-Apo 20× 0.8 NA or 63× 1.4 NA oil objective.

To analyze sessile hemocyte clusters in the larval cuticle, staged larvae expressing *UAS-G-TRACE* under the control of a GAL4 driver (Fig. 2, B and C) or larvae carrying *NRE-GFP* and *HmlΔ-DsRed* (Fig. 3 A) were pinned down in a Sylgard dissection dish containing ice-cold PBS and opened at the ventral side using Vannas spring scissors (Fine Science Tools). After removing the gut, central nervous system, and fat bodies, larval specimens were fixed in PBS containing 4% formaldehyde for 30 min and permeabilized in PBST-0.3 (PBS containing 0.3% Triton X-100) for 30 min. Larval specimens were incubated overnight with a mouse anti-Lz antibody (1:10, DSHB) diluted in PBST-0.3 containing 0.2% BSA at 4°C. Samples were incubated with a Cy5-conjugated donkey anti-mouse secondary antibody diluted in PBST-0.3 containing 0.2% BSA for 1 h at 25°C and mounted using Vectashield antifade mounting medium (Vector Laboratories). For each sample, a *z*-stack of two-dimensional images was acquired using a Zeiss LSM 800 microscope equipped with a Plan-Apo 20× 0.8 NA objective. For lineage tracing experiments using *UAS-G-TRACE*, GFP^+^, RFP^+^, or Lz^+^ cells were counted using the Spot module of Imaris 9.5.1 software (Oxford Instruments). In other experiments, Lz^+^ cells were counted manually.

LG dissections and immunostaining were performed essentially as previously described (Evans et al., 2014), with minor modifications. Briefly, wandering third instar larvae were dissected in ice-cold PBS to isolate the LG along with the brain/mouth hook complex. Dissections were completed within 30 min, after which samples were fixed in PBS containing 4% formaldehyde for 30 min and permeabilized in PBST-0.4 (PBS containing 0.4% Triton X-100) for 30 min. Samples were then incubated overnight with mouse anti-Lz antibody (1:10, DSHB) diluted in PBST-0.4 containing 5% BSA at 4°C, followed by incubation with Cy5-conjugated donkey anti-mouse secondary antibody in PBST-0.4 containing 5% BSA for 2 h at 25°C. LGs were carefully separated from the brain–mouth hook complex in Vectashield antifade mounting medium prior to imaging. In some experiments, larvae expressing *UAS-G-TRACE* (Fig. 2 F) under the control of *HmlΔ-GAL4* were used. For each sample, a *z*-stack of two-dimensional images was acquired using a Zeiss LSM 800 microscope equipped with a Plan-Apo 20× 0.8 NA objective. For lineage tracing experiments using *UAS-G-TRACE*, GFP^+^, RFP^+^, or Lz^+^ cells were counted using the Spot module of Imaris 9.5.1 software (Oxford Instruments). In other experiments, Lz+ cells were counted manually.

PI staining was performed by incubating peripheral hemocytes and LGs in Schneider’s medium with 30 μM PI, as previously described (Klemm et al., 2021).

### Endocytosis assays

Internalization of fluorescent endocytic tracers in primary hemocytes was quantitatively analyzed at 25°C as previously described (Kim et al., 2017). Briefly, primary hemocytes were collected by vortexing five third instar larvae with glass beads for 2 min and bleeding them in 50 μl of Schneider’s medium (Gibco) on a poly-L-lysine (PLL)–coated coverslip. Cells were allowed to settle on the coverslip for 30 min and then pulsed with 10 μg/ml Alexa Fluor 555–conjugated mBSA (Invitrogen), 2 mg/ml FITC-conjugated 70 kDa dextran (Dex70, Molecular Probes), or 1 mg/ml FITC-conjugated 10 kDa dextran (Dex10, Molecular Probes) in Schneider’s medium containing 1.5 mg/ml BSA for 5 min. After a 5-min chase in Schneider’s medium, cells were vigorously washed with chilled medium and fixed in PBS containing 4% formaldehyde for 10 min. Cells were then incubated in PBS containing 1 μg/ml DAPI for 10 min to stain nuclear DNA and mounted using Vectashield antifade mounting medium (Vector Laboratories). For each cell, a *z*-stack image was obtained using a Zeiss LSM 800 microscope equipped with a Plan-Apo 63× 1.4 NA oil objective. To quantify tracer internalization, total fluorescence per cell was measured by integrating intracellular fluorescence on all z-stack planes after correcting for background fluorescence and normalized to the total intensity of DAPI. ZEN 3.8 software (Zeiss) was used for image quantification.

For the Notch internalization assay, S2N cells transfected with *abi*, *Chc*, or *Rabankyrin* dsRNA were pretreated with M3 medium containing 0.7 mM CuSO_4_ for 24 h to induce Notch expression. Pretreated S2N cells (2.5 × 10^5^ cells) were plated with S2S cells (2.5 × 10^5^ cells) on a PLL-coated coverslip in 1 ml of serum-free M3 medium. Live cocultured cells were incubated in serum-free M3 medium at 4°C with 5 μg/ml mouse anti-NECD antibody (C458.2H; DSHB) for 30 min to label surface Notch receptors. They were then further incubated in M3 medium at room temperature for 30 min to allow internalization of labeled surface receptors. Following fixation without permeation, Notch receptors remaining on the surface of S2N cells were stained with a FITC-conjugated anti-mouse secondary antibody. Cells were subsequently permeabilized in PBST-0.2, and internalized Notch receptors in S2N cells and total Myc-Ser in S2S cells were stained with a Cy3-conjugated anti-mouse secondary antibody and a rabbit anti-Myc primary antibody plus a Cy5-conjugated anti-rabbit secondary antibody, respectively. An optical section through the middle of each cell was obtained using a Zeiss LSM 800 microscope equipped with a Plan-Apo 63× 1.4 NA oil objective. The total fluorescence intensities of surface (green) and internal (magenta) Notch receptors were measured using ImageJ, and the ratio of mean internalized to surface fluorescence intensities was used as the internalization index.

### Hemocyte counting and live imaging

To count embryonic crystal cells, WT and *abi*^*5*^/*Df* embryos carrying *UAS-mCD8-GFP* and *lz-GAL4* were collected on grape juice agar plates, dechorionated in 50% bleach, and fixed in a 1:1 mixture of 4% formaldehyde/0.05 M EGTA/PBS and heptane, as previously described (Lee et al., 2000). After sequential washing with methanol and PBS, embryos were mounted using SlowFade medium (Invitrogen). Stage 17 embryos were selected based on gut morphology, and *z*-stack images were acquired using a Zeiss LSM 800 microscope equipped with a Plan-Apo 20× 0.8 NA objective. GFP^+^ cells in the head mesoderm were manually counted.

To quantitatively analyze total peripheral hemocytes, late-third instar larvae were vortexed with glass beads for 2 min to release sessile hemocytes while leaving the LG intact, and then bled in 10 μl of Schneider’s medium (Gibco) on parafilm, as previously described (Petraki et al., 2015). The sample was transferred to a Neubauer improved hemocytometer (Marienfeld) for counting under an Olympus CKX53 microscope. In some experiments, total peripheral hemocytes from third instar larvae were stained with DAPI and an anti-P1, anti-Lz, or anti-L1 antibody. The percentage of plasmatocytes/crystal cells/lamellocytes was calculated by dividing the number of P1^+^/Lz^+^/L1^+^ cells by the total number of DAPI-stained hemocytes. Cells were imaged using a Zeiss LSM 800 microscope equipped with a Plan-Apo 20× 0.8 NA objective. P1^+^/Lz^+^/L1^+^ cells were manually counted. To quantitatively analyze sessile crystal cells, late-third instar larvae were heated at 70°C for 10 min. Larvae were imaged using an Olympus BX51 microscope equipped with an UPlanFL N 4× 0.13 NA objective, and blackened crystal cells were manually counted in the two most posterior dorsal segments (A7 and A8), where most crystal cells are sessile (Frankenreiter et al., 2021).

For live imaging, third instar WT and *abi*^*5*^/*Df* larvae carrying *HmlΔ-DsRed* were washed with ice-cold PBS and immobilized by cold treatment on a glass slide. Images were captured with an Axio Imager D1 fluorescence microscope equipped with an EC Plan-Neofluar 2.5× 0.085 NA objective.

### Statistical analysis

Data are presented as the mean ± standard error of the mean (SEM) of at least three independent experiments. Statistical significance was determined by the unpaired two-tailed Student’s *t* test or a one-way analysis of variance followed by post hoc pairwise comparisons of means using the Tukey–Kramer test. Data distribution was assumed to be normal, but this was not formally tested.

### Online supplemental material

Fig. S1 shows that loss of Abi does not affect total hemocyte numbers in third instar larvae or primary LGs, nor does it alter the abundance of crystal cells in embryos or second instar larvae. Fig. S2 shows that Abi is required for the maintenance of crystal cells in the LG. Fig. S3 shows that Abi-dependent CME is required for ligand-induced activation of Notch signaling. Fig. S4 shows the analysis of the transgenic expression of HA-Abi and its variants in Hml^+^ hemocytes. Fig. S5 shows opposing and independent regulation of hemocyte homeostasis by Abl and PTP61F.

## Supplementary Material

SourceData F6 is the source file for Fig. 6.

## Data Availability

The data that support the findings of this study are available from the corresponding author upon request.
