# Supplementary figures and images for: Drosophila Abi maintains blood cell homeostasis by promoting clathrin-mediated endocytosis of Notch
Source: J Cell Biol. 2025 Dec 26;225(3):e202505091. doi: 10.1083/jcb.202505091 (PMC12755901; doi:10.1083/jcb.202505091)

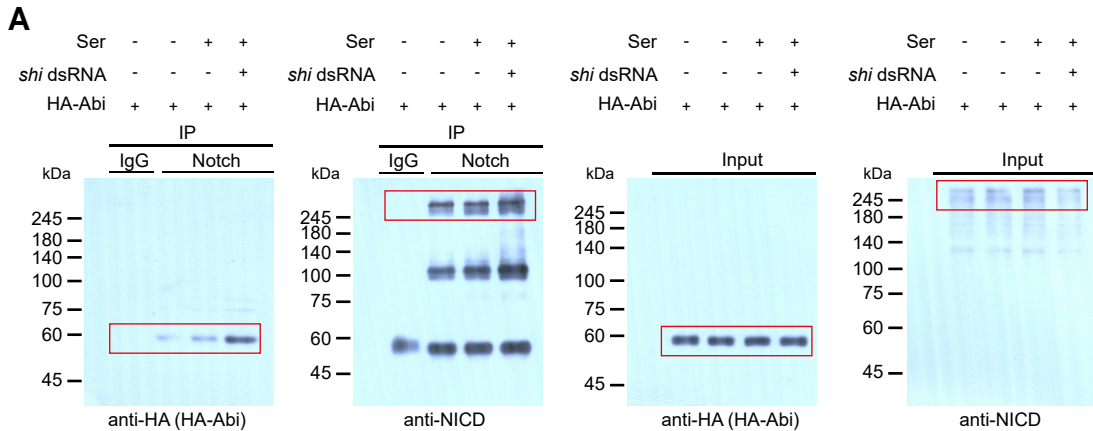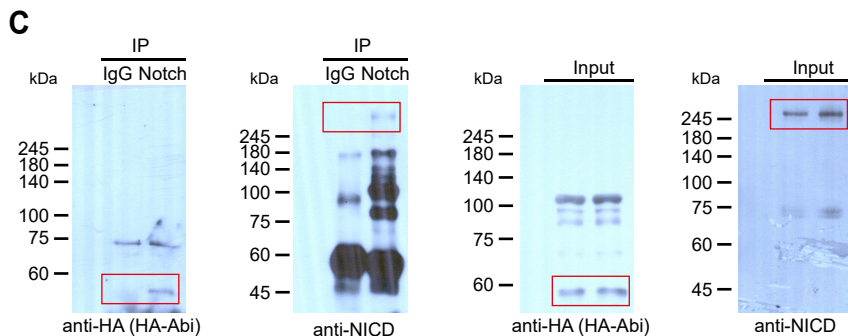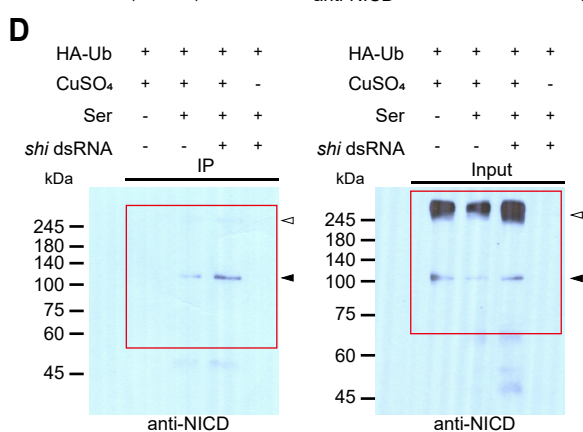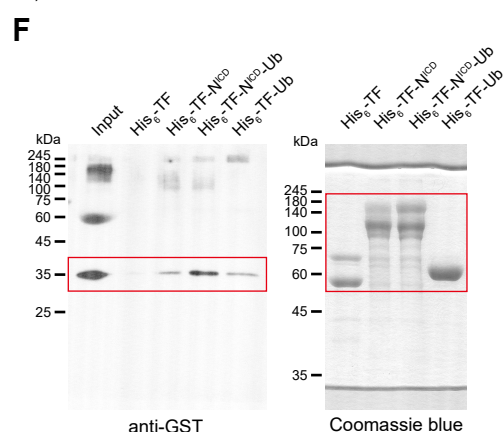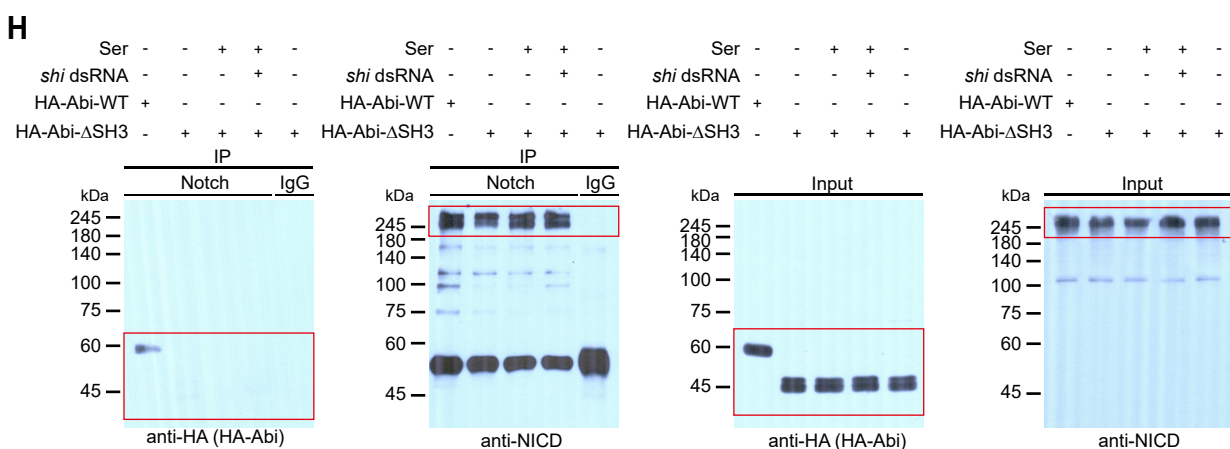

Supplement: SourceData F6 — is the source file for Fig. 6. [file jcb_202505091_sourcedataf6.pdf]
